# Unsupervised Learning Methods for Molecular Simulation Data

**DOI:** 10.1021/acs.chemrev.0c01195

**Published:** 2021-05-04

**Authors:** Aldo Glielmo, Brooke E. Husic, Alex Rodriguez, Cecilia Clementi, Frank Noé, Alessandro Laio

**Affiliations:** # International School for Advanced Studies (SISSA) 34014 Trieste, Italy; ‡ Freie Universität Berlin, Department of Mathematics and Computer Science, 14195 Berlin, Germany; § Freie Universität Berlin, Department for Physics, 14195 Berlin, Germany; ∥ Rice University Houston, Department of Chemistry, Houston, Texas 77005, United States; ⊥ International Centre for Theoretical Physics (ICTP), Condensed Matter and Statistical Physics Section, 34100 Trieste, Italy

## Abstract

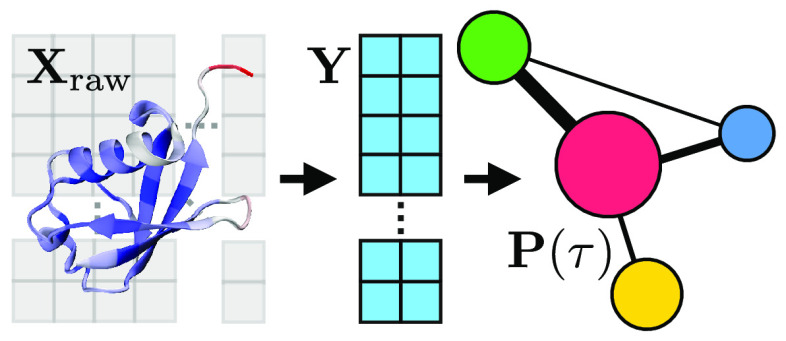

Unsupervised learning is becoming an essential tool to analyze the increasingly large amounts of data produced by atomistic and molecular simulations, in material science, solid state physics, biophysics, and biochemistry. In this Review, we provide a comprehensive overview of the methods of unsupervised learning that have been most commonly used to investigate simulation data and indicate likely directions for further developments in the field. In particular, we discuss *feature representation* of molecular systems and present state-of-the-art algorithms of *dimensionality reduction*, *density estimation*, and *clustering*, and *kinetic models*. We divide our discussion into self-contained sections, each discussing a specific method. In each section, we briefly touch upon the mathematical and algorithmic foundations of the method, highlight its strengths and limitations, and describe the specific ways in which it has been used-or can be used-to analyze molecular simulation data.

## 1 Introduction

In recent years, we have witnessed a substantial expansion in the amount of data generated by molecular simulation. This has inevitably led to an increased interest in the development and use of algorithms capable of analyzing, organizing, and eventually, exploiting such data to aid or accelerate scientific discovery.

The data sets obtained from molecular simulations are very large both in terms of the number of data points—namely, the number of saved configurations along the trajectory—and in terms of the number of particles simulated, which can be several millions or more. However, we know both empirically and from fundamental physics that such data usually have much lower-dimensional representations that convey the relevant information without significant information loss. A striking example is given by the kinetics of complex conformational changes in biomolecules, which, on long time scales, can be well described by transition rates between a few discrete states. Moreover, symmetries, such as the invariance of physical properties under translation, rotation, or permutation of equivalent particles, can also be leveraged to obtain a more compact representation of simulation data.

Traditionally, finding such low-dimensional representations is considered a task which can be tackled based on domain knowledge: The analysis of molecular simulations is often performed by choosing a small set of *collective variables* (CVs), possibly complex and nonlinear functions of the coordinates, that are assumed to provide a satisfactory description of the thermodynamic and kinetic properties of the system. If the CV is appropriately chosen, a histogram created from the CV summarizes all the relevant information, even if the molecular system includes millions or billions of atoms. The list of possible CVs among which one can choose is enormous and is still growing. Tools now exist which can describe phenomena of high complexity, such as the packing of a molecular solid, the allostery of a biomolecule, or the folding path of a protein. However, choosing the right CV remains to some extent an art, which can be successfully accomplished only by domain experts, or by investing a significant amount of time in a trial-and-error procedure.

Machine learning (ML) has emerged as a conceptually powerful alternative to this approach. ML algorithms can be broadly divided in three categories.^1^ In *supervised* learning, a training data set consisting of input–output pairs is available, and a ML algorithm is trained with the goal of providing predictions of the desired output for unseen input values. This approach has been extensively used to predict specific properties of materials and molecules, such as the atomization energy of a compound or the force acting on an atom during a trajectory. In *unsupervised* learning, no specific output is available for the data in the training set, and the goal of the ML algorithm is to extract useful information using solely the input values. A typical application of this approach in the field of molecular simulation is the construction of low dimensional collective variables, which can compactly yet effectively describe a molecular trajectory. Finally, in *reinforcement* learning, no data at all is used to train the ML model, which instead learns by “trial and error” and by continuously interacting with its environment. Reinforcement learning has proven particularly successful at certain computer science challenges (such as playing board games), and it is now beginning to find application in molecular and materials science. In this Review, we focus exclusively on unsupervised learning methods. We aim to provide an overview of all algorithms currently used to extract simplified models from molecular simulations to understand the simulated systems on a physical level. The review is oriented toward researchers in the fields of computational physics, chemistry, materials and molecular science, who routinely deal with large volumes of molecular dynamics simulation data, and are hence interested in using, or extending, the techniques we describe.

Some of the algorithms we review are as old as the simulation methods themselves, and have been successfully used for decades. Others are much more recent and their conceptual and practical power is only now becoming clear. Despite the recent surge in, for example, machine learning algorithms for determining CVs, many problems in the field can still be considered open, making this research area extremely active.

**Figure 1:**
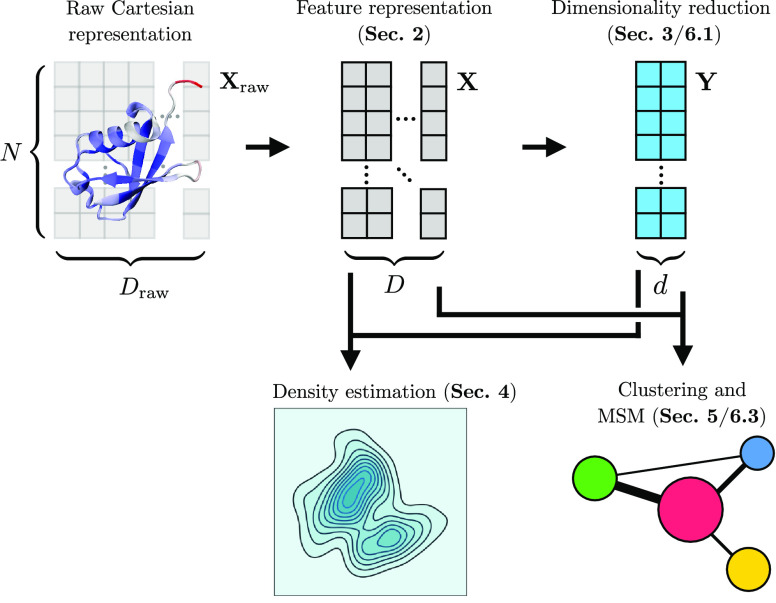
Illustration of the possible steps that can be performed to analyze data from a molecular simulation with an indication of the particular section where these are discussed.

We divide our discussion in five sections, each of which will include its own introduction section: *feature representation*, *dimensionality reduction*, *density estimation*, *clustering*, and *kinetic models*. A graphical table of contents depicting the different methods and how they relate to each other is shown in Figure 1.

In Section 2, we discuss the choice of feature representation for atomistic and molecular systems, a topic relevant for any analysis or application of learning algorithm. We, then, turn to the description of unsupervised learning algorithms, which we divide in four groups. In Section 3, we review algorithms of *dimensionality reduction*, whose primary objective is to provide a low dimensional representation of the data set that is easy to analyze or interpret. While in molecular simulations the probability density from which the configurations are harvested is in principle known (e.g., the Boltzmann distribution), this probability density is defined over the whole molecular state space, which is too high-dimensional to be visualized and understood. In Section 4, we review algorithms of *density estimation*, which enable the estimation of probability distributions restricted to sets of relevant variables describing the data. These variables can be chosen, for example, using the techniques described in Section 3. In Section 5, we review *clustering* algorithms. Clustering divides the data set into a few distinct groups, or “clusters,” whose elements are similar according to a certain notion of distance in their original space. These techniques allow to capture the gross features of the probability distribution; for example, the presence of independent probability peaks, even without performing a preliminary dimensionality reduction. Therefore, the techniques described in this section can be considered complementary to those described in Section 3. When clustering is viewed as an assignment of data points to integer labels, clustering itself can be viewed as an extreme form of dimensionality reduction. In Section 6, we present *kinetic models*. While Sections 3-5 focus on modeling methods in which the ordering of the data points is not considered, kinetic models instead exploit dynamical information (i.e., ordering of data points in time) to reduce the dimensionality of the system representation. Altogether, this set of approaches is qualitatively based on the requirement that a meaningful low-dimensional model should reproduce the relevant time-correlation properties of the original dynamics (e.g., the transition rates). Throughout the review we present these techniques highlighting their specific application to the analysis of molecular dynamics, and discussing their advantages and disadvantages in this context. In Section 7 we list the software programs which are most currently used to perform the different unsupervised learning analysis described. Finally, in the Conclusions (Section 8), we present a general perspective on the important open problems in the field.

Some other review articles have a partial overlap with the present work. In particular, ref (2) reviews algorithms of dimensionality reduction for collective variable discovery, while refs (3 and 4) also review some approaches to build kinetic models. In this work, we review not only all these approaches but also other algorithms of unsupervised learning, namely, density estimation and clustering, focusing on the relationship between these different approaches and on the perspectives opened by their combination. Other valuable review articles of potential significance to the reader interested in machine learning for molecular and materials science are ref (5−9).

## 2 Feature Representation

Throughout this Review, we assume to have obtained molecular data from a simulation procedure, such as Monte Carlo (MC)^10^ or molecular dynamics (MD).^11^ In general, the data set comes in the form of a *trajectory*: a series of *configurations*, each containing the positions of all particles in the system. These particles usually correspond to atoms but may also represent larger “sites”; for example, multiple atoms in a coarse graining framework. Given a trajectory data set, before any machine learning algorithm can be deployed, we must first choose a specific numerical representation **X** for our trajectory. This amounts to choosing a set of “features” (often referred to as “descriptors” or “fingerprints”) that adequately describes the system of interest.

The “raw” trajectory data set (the direct output of the simulation procedure) is represented by a matrix , where r stands for “raw”, *N* is the number of simulation time points collected, and *D*_r_ is the number of degrees of freedom in the data set. When three-dimensional spatial coordinates are retained from the simulation, *D*_r_ is equal to three times the number of simulated particles. When momenta are also retained, *D*_r_ is equal to six times the number of simulated particles. It is from this representation that we seek to featurize our data set; namely, transform our data into a new matrix . For later reference, we denote the function that performs the featurization on each “raw” data point **x**_*i*,r_ as , that is,
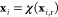
1A trivial featurization is to let **X** = **X**_r_ (and thus *D* = *D*_r_). In this case, the “raw” data output of the simulation is directly used for analysis. For molecular systems, however, this is often disadvantageous, since *D*_r_ is typically very large.

When sufficient prior knowledge of the system is available, it is convenient to choose a feature space such that *D* ≪ *D*_r_ that can appropriately characterize the molecular motion of interest without significant loss of information. Often, the number of degrees of freedom required to encode many physical properties or observables of a molecular system is relatively low.^12,13^ In the following, we will review features that have been frequently used to represent atomic and molecular systems. We can divide these features into two fundamentally different groups, which we will discuss in turn. For certain physical systems, such as biomolecules, each atom may be considered as having a unique identity that represents its position in a molecular graph. For such systems, it is often convenient to use features that *are not* invariant with respect to permutations of chemically identical atoms. On the other hand, for most condensed matter systems, and materials, atoms of the same element should be treated as indistinguishable, and descriptors should be invariant under any permutation of chemically identical atoms. We conclude this section with a brief discussion of representation learning, a promising new direction for determining features automatically.

It is worth noticing here that an explicit numerical representation for the molecular trajectory is not always necessary since some algorithms can work even if only a distance measure between molecular structures is defined.^14−22^ The main limitation of this approach is that its computational cost scales quadratically with the number of data points.

### 2.1 Representations for Macromolecular Systems

Many of the methods discussed in this Review have been applied to classical simulation data sets of systems such as DNA, RNA, and proteins. A macromolecular simulation of such a system will contain at least one solute (for example, a protein) comprising 100–100 000 atoms connected by covalent bonds which cannot be broken. The system will also typically include several thousand water molecules, as well as several ions and lipids. Other small molecules, for example a drug or other ligand, are often also included.

In most analyses of macromolecules, the focus is on the solute and the solvent degrees of freedom are neglected. In the absence of an external force, we then expect the solute’s dynamics and thermodynamics to be invariant to translation and rotation of the molecule (in three dimensions for fully solvated systems and in two dimensions for membrane-bound systems). A simple manner of obtaining a set of coordinates obeying this invariance is to remove the solvent molecules and, then, superpose (i.e., align) each configuration in the trajectory to a reference structure of the molecule (or of the complex), which is known to be of physical or biological interest. This can be done by finding the rotation and the translation minimizing the root-mean-square deviation (RMSD) with the reference structure.^23^

While in many cases, this simple procedure is adequate, in many others it is suboptimal; for example, when a reference structure is not known or when the configurations are too diverse to be aligned to the same structure. This is the case for simulations of very mobile systems, such as RNA and intrinsically disordered proteins, or for the simulation of folding processes, in which the system dynamically explores extremely diverse configurations that cannot be meaningfully aligned to a single structure. Beyond RMSD-based approaches, an appropriate choice of features satisfying rotational and translational invariance are the so-called internal coordinates, first introduced by Gordon and Pople.^24^ For classical simulations of proteins or nucleic acids, one can consider the bond lengths and the angles formed by three consecutive atoms as approximately fixed, and therefore, the configuration of the molecule can be described by the value of the dihedral angles formed by four consecutive atoms.^25,26^ For example, in a protein the configuration of the backbone is defined by the so-called ϕ- and ψ-dihedral (Ramachandran) angles.^27^ Using these coordinates, which by construction are invariant with respect to system rotation and translation, enables a significant dimensionality reduction of the system’s representation. For example, an amino acid residue has six backbone atoms, which corresponds to 18 degrees of freedom if one uses their Cartesian coordinates to represent the residue. On the other hand, their position is determined with good approximation by the value of only the two Ramachandran angles.

For larger systems whose dynamics are characterized by more global changes in conformation (as in protein folding or allostery), it is more common to use a contact-based featurization. For example, the “contact distances” between all monomers can be used according to a predesignated site (e.g., α-carbons in a protein) or the closest (heavy) atoms of each pair. These contacts can be also defined by selecting a given cutoff distance and then using a contact indicator function.^25^

There are many more specialized feature representations that can be employed according to the problem under study. For example, in simulations that involve two or more solute molecules, such as ligand binding or protein–protein association, distances, angles, and other data specific to the binding site can be used.^28,29^ In some cases, featurizations involving assignment to secondary structure elements,^30^ solvent-accessible surface area,^31^ or hand-picked features may be most appropriate.^32^ Furthermore, different types of features can be concatenated (and appropriately scaled). In some cases, featurizations that explicitly or implicitly treat solvent molecules, lipids or ions are important. Examples include when characterizing the solvent dynamics around binding pockets,^33^ ions moving through ion channels,^34,35^ or the lipid composition around membrane proteins.^36,37^ Several publications have compared the suitability of various feature sets for protein data for analyses, particularly in the context of the kinetic models described in Section 6.^38−40^

### 2.2 Representations for Condensed Matter Systems

In many condensed matter systems, such as solvents and materials, the physical properties of interest are invariant with respect to the exchange of equivalent atoms or molecules as such permutations merely exchange the particle labels. In such situations, it is often essential that the system configurations are represented in a permutation-invariant way. For example, in a pure water simulation, water molecules diffuse around and change places. Comparing different configurations by a simple RMSD, which depends on the order of molecules in the coordinate vector, is therefore inadequate.

A direct approach for working with Cartesian coordinates in a way that preserves permutational invariance is to relabel exchangeable particles or molecules such that the distance to a reference configuration is minimized. For example, in a simulation of water, one could define an ice-like configuration where water molecules are aligned on a lattice as a reference configuration. The (arbitrary) sequence in which waters are enumerated in this configuration defines the reference labeling. Then, for every configuration visited during the simulation, one must determine to which label each water molecule should be assigned (i.e., which permutation matrix should be applied) such that the relabeled configuration will have the minimal RMSD to the reference configuration. This problem can be solved by a bipartite matching method, such as the Hungarian algorithm.^41^ Such approaches have been applied to estimate solvation entropies^42^ and sampling permutation-invariant system configurations.^43^

Another common strategy for designing permutation-invariant features is to first represent a system as a union of low order constituents (“*n*-plets”) for which a permutation-invariant descriptor is computed. In this approach, invariance can be achieved by enumerating all permutations of subsets of permutable particles or molecules. While this is not possible for permutations (the number of which scales factorially), it is relatively straightforward for pairs or triplets of atoms.^44−46^ As a simple example, a system of *M* atoms is first broken down into the *M*(*M* – 1)/2 unique pairs of atoms that constitute it. Then, the distribution functions of interatomic distances between two atomic species is computed. Finally, these distribution functions are binned to produce permutation invariant feature vectors.

Different descriptors have been proposed which build upon the above idea in the context of specific aims. For example, Atom-centered symmetry functions (ACSFs)^47,48^ are obtained by expanding 2-body (radial) and 3-body (angular) distribution functions onto specific invariant bases. The ACSF representation was the first widely adopted representation for materials systems. ACSFs are very efficient to evaluate numerically and they have been shown to be able to resolve geometric information well enough to train highly efficient interatomic potentials.^49,50^ ACSFs are now widely used in shallow neural networks that replace molecular force fields by learning quantum-chemical energies.^47,48,51,52^

Another commonly used descriptor for materials system is the so-called Smooth Overlap of Atomic Positions (SOAP).^53,54^ The SOAP representation is obtained by expanding a smoothed atomic density function onto a radial and a spherical harmonics basis set. This representation can be considered an expansion of 3-body features.^55^ The SOAP vector has also been shown to resolve geometric information very precisely.^54^

Many other representations based on the *n*-plets principle have been suggested. They differ in the specific low-order contributions chosen and in the particular basis in which the low order information is expanded. Notable examples are the Coulomb matrix,^56^ the “Bag of Bonds”,^57^ the histograms of distances, angles, and dihedral angles (HDAD),^58^ and the many body tensor representation (MBT).^59^ In general, finding informative numerical representations for condensed matter systems is a very hard problem,^60,61^ and an exhaustive review of all the approaches which have been proposed to tackle it is beyond the scope of this work. The interested reader can refer to refs (62−64) for a more comprehensive analysis of the different descriptors developed for systems with permutation invariance.

### 2.3 Representation Learning

In the past few years, substantial research has been directed toward learning a feature representation rather than manually selecting it. Good examples of representation learning algorithms for molecules are given by graph-neural networks (GNNs)^65^ such as SchNet,^66^ PhysNet,^67^ DimeNet,^68^ Cormorant,^69^ or Tensor field Networks.^70^ These networks are usually trained to predict molecular properties, and they do so by performing continuous convolutions across the spatial neighborhoods of all atoms. In contrast to other neural network approaches based on fixed representations,^47,71^ in GNNs the representations are not predefined but are instead learnable using convolutional kernels. The feature representation is a vector attached to each atom or particle. It is initialized to denote the chemical identity of the particle (e.g., nuclear charge or type of bead), and it is then updated in every neural network layer depending on the chemical environment of every particle and through the action of convolutional kernels whose weights are optimized during training. The representation found at the last GNN layer can thus be interpreted as a learned feature representation which encodes the many-body information required to predict the target property (e.g., the potential energy of the molecule).

GNNs have been extensively used to predict quantum-mechanical properties^66−68,72^ and coarse-grained molecular models.^73,74^ See refs (4 and 75) for recent reviews of these topics.

For the rest of this Review, we will assume that the featurization step has been performed; and a sufficiently general numerical representation **X**, appropriate for the system under study, has been obtained.

## 3 Dimensionality Reduction and Manifold Learning

In this section, we review dimensionality reduction techniques that have been used most extensively in the analysis of molecular simulations. All these techniques involve transforming a data matrix **X** of dimensions *N* × *D* into a new representation **Y** of dimensions *N* × *d*, with *d* ≪ *D*, with the goal of preserving the information contained in the original data set. It is typically impossible to achieve this task exactly, and the different methods reviewed here can only provide different approximate solutions whose utility needs to be evaluated on a case by case basis. We first describe linear projection methods: Principal component analysis and Multidimensional scaling. These are arguably the simplest and most widely used techniques of dimensionality reduction. However, if the data do not lie on hyperplanes these linear methods can easily fall short. In the subsequent sections we describe a set of nonlinear projection methods that can deal also with data lying on twisted and curved manifolds. In order, we will cover Isomap, Kernel PCA, Diffusion map, Sketch-map, t-SNE, and deep learning methods.

We will present the theory beyond the mentioned algorithms only briefly; the interested reader can refer to existing specialized reviews for more information on such aspects.^3,76,77^ Instead, we will focus our attention on the practical issues related to utilizing these methods for the analysis of molecular trajectories and highlight relative merits and pitfalls of each method.

**Figure 2:**
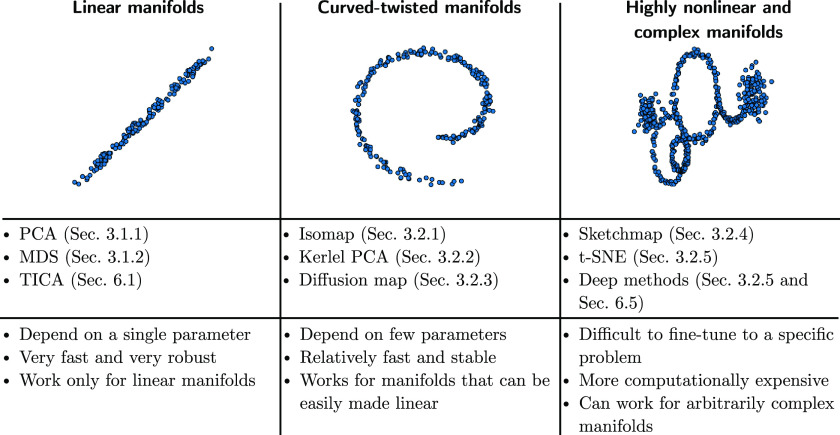
Different algorithms of dimensionality reduction discussed, divided in three groups. From left to right, the listed algorithms are capable of dealing with manifolds of higher complexity, which comes at the price of a higher computational cost and a larger number of free parameters to choose.

The mentioned algorithms are grouped together for reference in Figure 2. The figure emphasizes that a manifold of increasing complexity can only be efficiently reduced in dimensionality using methods of increasing sophistication, which however will typically require a greater computational power and a longer trial and error procedure to be properly deployed.

### 3.1 Linear Dimensionality Reduction Methods

#### 3.1.1 Principal Component Analysis

**Figure 3:**
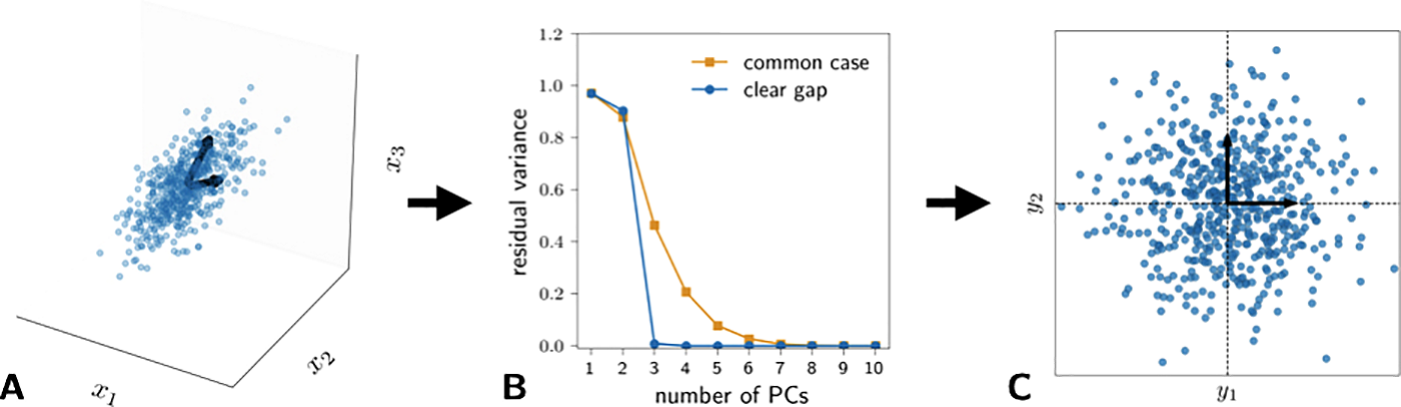
Illustration on the working principle of the PCA projection. (A) Two-dimensional linear manifold embedded in a high dimensional space. (B) Eigenspectrum of a covariance matrix of the data, as the manifold is two-dimensional a clear gap appears after the second eigenvalues (blue line); a more typical eigenspectrum is shown in orange. (C) Low-dimensional representation of the data obtained through PCA.

Principal component analysis (PCA)^78−80^ is possibly the best known procedure for reducing the dimension of a data set and further benefits from an intuitive conceptual basis. The objective of PCA is to find the set of orthogonal directions along which the variance of the data set is the highest. These directions can be efficiently found as follows. First, the data is centered by subtracting the average, obtaining a zero-mean data matrix **X̂**. This operation guarantees the translational invariance of the PCA projection. Second, the covariance matrix of the data is estimated as . Finally, the eigenvectors of the matrix **C** are found by solving the eigenproblem
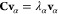
2It is simple to show that the direction of maximum variance coincides with **v**_1_, the eigenvector corresponding to the largest eigenvalue λ_1_.^80^ The direction of maximum variance in the subspace orthogonal to **v**_1_ is **v**_2_, and so on. The PCA representation is ultimately obtained by choosing a number of components *d*, and projecting the original data onto these vectors as **Y** = **XV**, where **V** is a matrix of dimension *D* × *d* containing the first *d* eigenvectors of **C**. An illustration of this procedure is shown in Figure 3a on a data set obtained by harvesting data points from an 10-dimensional Gaussian stretched in two dimensions.

Importantly, the eigenvalue *λ*_*α*_ is exactly equal to the variance of the data along the given direction **v**_α_.^80^ For this reason, it is common to select the dimensionality *d* most appropriate for a PCA projection by looking at the eigenvalue spectrum as a function of the eigenvalue index. A clear gap in such a plot, as seen in the blue curve in Figure 3b, is an indication that a dimensionality reduction including the components before the gap is meaningful. In real world applications, however, it is common to observe a continuous, although fast, decay of the magnitude of the eigenvalues (orange curve in Figure 3b). In this situation the selection of *d* is more arbitrary. A common rule of thumb is to choose the smallest *d* that is able to capture a good portion of the total variance of the data set. In practice, one can choose the smallest *d* such that Σ_*i*=α_^*d*^*λ*_*α*_/Σ_*i*=α_^*D*^*λ*_*α*_ ≥ *f*, where *f* is a free parameter, which is often set to 0.98 or 0.95.

The use of PCA to analyze biomolecular trajectories was first proposed in ref (81−83). In these papers, it was numerically found that a very small fraction of coordinates were capable of describing the majority of the motion of the molecules studied. These coordinates have often been named *essential coordinates* and, consistently, the methodology has been named “essential dynamics”.^83^ Importantly, the variation along the essential degrees of freedom was connected to the functional motion of the protein. In the years that followed, PCA has been extensively used to characterize both functional motions and the free energy surface of small peptides and proteins.^84−90^ In ref (91), the use of PCA was also proposed in conjunction with metadynamics for enhanced sampling. In the context of solid state and materials physics, PCA has been commonly used for exploratory analysis, visualization, data organization, and structure–property prediction.^92−94^

In spite of its empirical success, the theoretical foundation of the use of PCA for the analysis of molecular trajectories has been the subject of debate. In particular, soon after essential dynamics was proposed it was argued that the sampling needed to robustly characterize the essential coordinates was beyond the time-scales accessible to MD simulations.^95,96^ Other studies, however, argued that the convergence time needed for the characterization of a stable eigenspace of principal components is reachable and in the range of nanoseconds of simulation time.^97−100^ Aside from sampling concerns, the strong assumption of the existence of a linear manifold which correctly captures the important modes of variation of a molecular system can easily fall short, giving rise to systematic errors in analysis and predictions.^101^ The approach presented in the next section provides a manner for overcoming this limitation.

#### 3.1.2 Multidimensional Scaling

Multidimensional scaling (MDS)^102,103^ is closely related to PCA, and equivalent to it under certain conditions. MDS will be the basis for the advanced nonlinear dimensionality reduction techniques which will be described in the following sections. MDS provides a low dimensional description of the data by finding the *d* dimensional space which best preserves the pairwise distances between points. It does so by minimizing a cost function quantifying the difference between the pairwise distance *R*_*ij*_ as measured in the original *D* dimensional space and the one computed in the low dimensional embedding
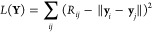
3If the distance matrix is given by *R*_*ij*_ = ∥**x**_*i*_ – **x**_*j*_∥, then, it is simple to show that the vectors **y**_α_ that minimize eq 3 are found solving the following eigenproblem^104^
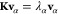
4and taking . The matrix **K** in eq 4 contains the inner products of all the centered data vectors *K*_*ij*_ = **X̂**_*i*_^T^**X̂**_*j*_, where . The key trick of MDS is that such a matrix can be obtained from the matrix of distances *R*_*ij*_ = ∥**x**_*i*_ – **x**_*j*_∥ in a simple way, namely^104,105^
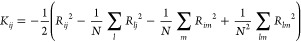
5It is important to note that the embeddings generated by MDS and PCA are *exactly equivalent* if the distance between the data points is estimated as *R*_*ij*_ = ∥**x**_*i*_ – **x**_*j*_∥. This follows from the fact that the eigenvectors of the covariance matrix {**v**_α_^*c*^} and of those of the **K** matrix {**v**_α_^*k*^} are related to each other as .^76^

The covariance matrix **C** and the matrix of inner products **K** have dimensions *D* × *D* and *N* × *N*, respectively, which greatly affects the computational cost of the methods. So, for instance, if the number of points greatly exceeds the number of dimensions *N* ≫ *D*, PCA is more computationally efficient, while the contrary is true if *D* ≫ *N*.

The most important difference between PCA and MDS lies in the fact that MDS can be used also when the data matrix **X** is not available and one is only provided with the matrix *R*_*ij*_ of pairwise distances between points. This is an important feature of MDS, which allows it to also work in the context of nonlinear dimensionality reduction as we will describe in the next section.

### 3.2 Nonlinear Dimensionality Reduction

#### 3.2.1 Isomap

The Isometric feature mapping (Isomap) algorithm^106^ was introduced with the goal of alleviating the problems of linear methods, such as PCA, which fail to find the correct coordinates whenever the embedding manifold is not a hyperplane. The key idea of Isomap is the generation of a low dimensional representation that best preserves the *geodesic* distances between the data points on the data manifold, rather than the Cartesian distance.

**Figure 4:**
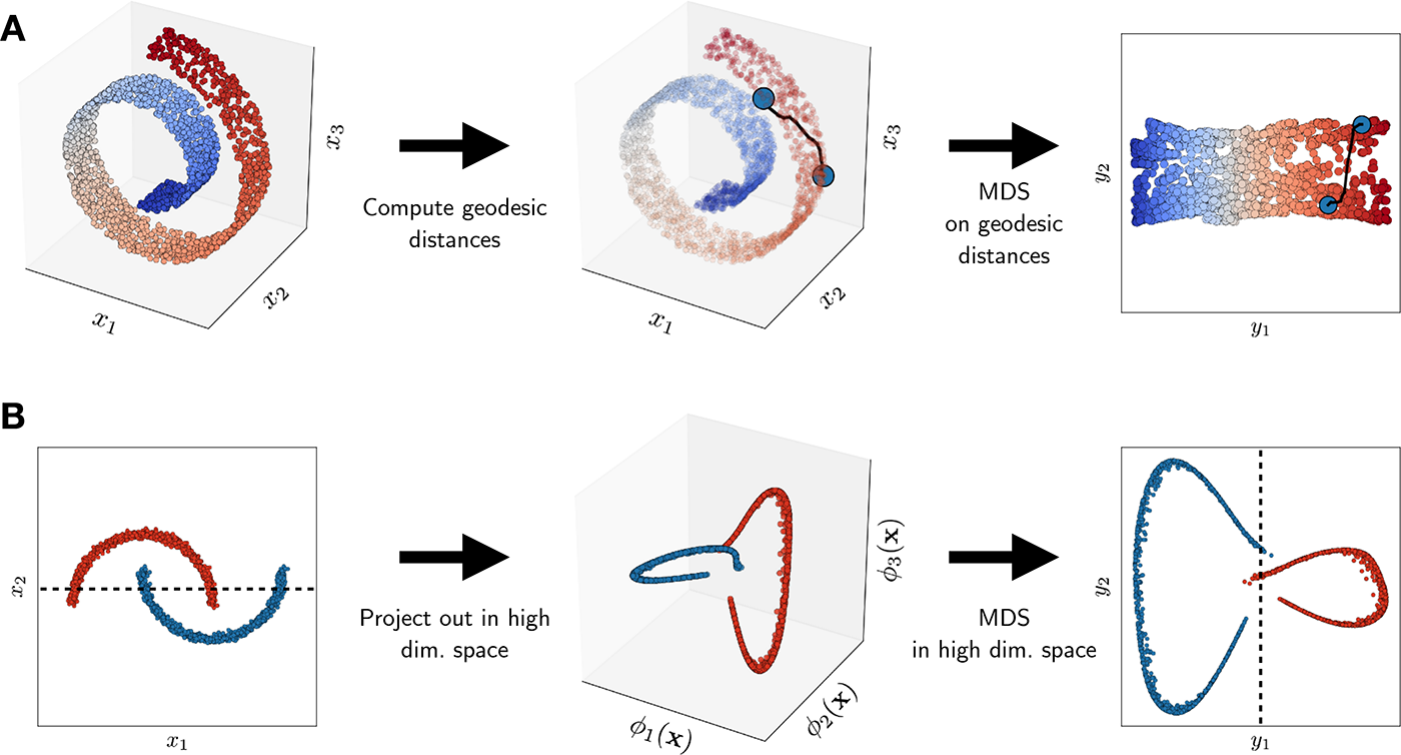
(A) Illustration of Isomap on a Swiss roll data set. The original 3D data set (left) is projected on a 2D space in a way that optimally preserves geodesic distances between points (middle and right). (B) Illustration of Kernel PCA on a data set with two segments that are not linearly separable. The original 2D data set (left) is first represented in a higher dimensional space, where it becomes linearly separable (middle), and finally, MDS is performed on the transformed data set (right). Note that the transformed data set typically lies in a space of very high—and often infinite—dimensionality, and the 3D embedding shown here only serves illustrative purposes.

Isomap comprises three steps. First, a graph of the local connectivities is constructed. In this graph, each point is linked to its *k*th nearest neighbors with edges weighted by the pairwise distances. Second, an approximation of the geodesic distance between all pairs of points is computed as the shortest path on this graph. Finally, MDS is performed on the matrix *R*_*ij*_ containing the geodesic distances. The final representation thus minimizes the loss function in eq 3, and provides the low dimensional Euclidean projection that best preserves the computed geodesic distances. An illustration of the working principle of Isomap is presented in Figure 4.

A drawback of Isomap is its potential for topological instabilities. Indeed, if the manifold containing the data is not isomorphic to a hyperplane (for example, it is isomorphic to a sphere or to a torus) the procedure becomes ill defined, since a sphere or a torus cannot be mapped to a hyperplane without cutting it. More generally, the quality of the representation generated depends on the quality of the geodesic distances, which can only be estimated approximately. In particular, the algorithm used to compute geodesic distances requires choosing which data points can be considered directly connected, namely close enough that their geodesic distance coincides with their Cartesian distance. Considering connected points which are too far apart can bring to a severe underestimation of the geodetic distance, if the manifold is strongly curved.^107^

The computation of the geodesic distance between all pairs of points is also the main performance bottleneck when using Isomap on large data sets of molecular trajectories. For this reason, when Isomap was first used for the analysis of molecular data sets,^101^ it involved the preselection of a small number of landmark points *n*_L_ that were assumed to correctly span the data manifold. This modified procedure, named “ScIMap” (Scalable Isomap), is much faster than the standard Isomap implementation since the geodesic distance is computed only between a small fraction *n*_L_ ≪ *N* of landmark points. The scalability of Isomap to large data sets was further improved with the introduction of “DPES-ScIMAP” (Distance-based Projection onto Euclidean Space ScImap) in ref.^108^ This procedure involves an initial projection of the points onto a low-dimensional Euclidean space.

In ref (101), it is shown that using Isomap in place of PCA allows describing the folding process of a small protein by much less variables. This result is confirmed in refs (109) and (110), in which it is shown that Isomap coordinates faithfully describe the motion of small molecules and the free energy landscape of small peptides. On the other hand, in ref (111), only small improvements were observed when using Isomap in place of PCA for describing the folding process of another peptide. In ref (112), an Isomap embedding was successfully used to generate the collective biasing variables for a metadynamics simulation. In ref (113), Isomap was employed to construct an enhanced MD sampling method.

In general it is clear that the extent to which a nonlinear method like Isomap proves beneficial—or even necessary—depends upon the degree of nonlinearity of the manifold in which the molecular trajectory is (approximately) contained). This nonlinearity depends on the system under study as well as on the type of coordinates chosen to describe it (see section 2). While it is difficult to determine precisely the degree of nonlinearity of a data manifold, an approximate estimate can be obtained by comparing its linear dimension, obtained for instance by an analysis of the PCA eigenspectrum (Figure 3B), with its nonlinear *intrinsic dimension*, which can be computed using several tools.^114,115^ An intrinsic dimension much smaller than the linear dimension can then be seen as an indication of the presence of curvature and topological complexity in the manifold. Providing a quantitative measure of this complexity can still be considered an open problem.

#### 3.2.2 Kernel PCA

A different strategy for finding a low-dimensional representation for data points embedded in a curved and twisted manifold is Kernel PCA.^116^ In Kernel PCA, data are represented in a new space using a nonlinear transformation **ϕ**(**x**), where **ϕ** is a high-dimensional vector-valued function. A linear dimensionality reduction is then performed in this space. The transformation **ϕ** should be chosen in such a way that even if the original data manifold is nonlinear, the transformed data set is approximately linear, allowing for the usage of MDS in the transformed space. An illustration of this concept is provided in Figure 4.

In Kernel PCA, the transformation is not performed explicitly, but obtained through the use of a kernel function κ(**x**, **x**′). By definition, a kernel function represents a dot product in some vector space. Hence, one sets
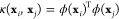
6which implicitly defines the function **ϕ**. One then implicitly removes the mean from the transformed data^105^ by the same procedure used in MDS (see eq 5)
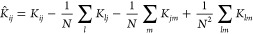
7where *K*_*ij*_ = κ(**x**_*i*_, **x**_*j*_). Finally, the MDS algorithm is used on such a matrix, providing the principal components of the data in the transformed space.

It is crucial to note that the transformed data matrix is never explicitly computed in the algorithm. One only needs to compute the matrix of dot products **K**, and this can be done efficiently through the use of the kernel function. This fact is known as the “kernel trick”,^1^ and it allows to transform the data in spaces of very high and often infinite dimensionality, thereby enabling the encoding of highly nonlinear manifolds without knowing suitable feature functions. A drawback, however, is that the kernel matrix has the dimension of *N*^2^, resulting in unfavorable storage and computing costs for large data sets.

The simplest kernel one can use is the linear kernel κ(**x**_*i*_,**x**_*j*_) = **x**_*i*_^T^**x**_*j*_, which makes Kernel PCA equivalent to standard PCA. Polynomial kernels of the kind κ(**x**_*i*_,**x**_*j*_) = (**x**_*i*_^T^**x**_*j*_)^*p*^ can increase the feature space systematically for larger values of the parameter *p*. The use of a specific kernel, allows recovering Isomap.^117^ Perhaps the most widely used kernel for Kernel PCA is the Gaussian kernel κ(**x**_*i*_,**x**_*j*_) = *e*^–||**x**_*i*_–**x**_*j*_||^2^/2*h*^2^^. The Gaussian kernel depends on the parameter *h*, which determines the length scale of distances below which two points are considered similar in the induced feature space.

Kernel PCA is a powerful method for nonlinear dimensionality reduction, since in principle it can overcome the limitations of linear methods without a significant increase in computational cost. However, a practical issue in using Kernel PCA lies in its sensitivity to the specific choice of kernel function used and any parameters it may have. In ref (118), it is suggested to choose the kernel by systematically increasing the kernel complexity (from the simple linear kernel to the polynomial kernel with *p* = 2 and so on) until a clear gap in the engenspectrum of the kernel matrix appears (see discussion of Figure 3b). Although this procedure is not guaranteed to be successful in general, in ref.^118^ the authors successfully identify the reaction coordinate of a protein with the aid of Kernel PCA and a polynomial kernel. We refer to ref.^119^ for more information on the use of Kernel PCA and the various possible choices of kernels to analyze molecular motion.

In the context of materials physics, Kernel PCA has been particularly fruitful when used in conjunction with the SOAP descriptors (reviewed in section 2.2). Indeed, SOAP descriptor forms the basis for accurate interpolators of atomic energies and forces,^120−122^ meaning that this descriptor generates data manifolds in which similar materials lie close to each other. Kernel PCA has been successfully used for visualization and exploration of materials databases,^123^ for identifying new materials candidates,^92^ and to predict phase stability of crystal structures.^124^

A common feature of PCA, MDS, and Isomap is that their result is strongly affected by the largest pairwise distances between the data points. This can be a problem as the distances deemed relevant for the analysis of molecular trajectories are often those of intermediate value: not the largest, which only convey the information that the configurations are different, and not the smallest, whose exact value is also determined by irrelevant details (for example bond vibrations). Kernel PCA can partially accommodate this issue via the choice of kernel, since the distances which are preserved are those computed in the transformed space that the kernel implicitly generates. In the next subsection, we will describe Diffusion map, another projection algorithm that can be used in these situations.

#### 3.2.3 Diffusion Map

Diffusion map^125,126^ is another technique similar to Kernel PCA, which enables the discovery of nonlinear variables capable of providing a low-dimensional description of the system. The diffusion map has a direct application to the analysis of molecular dynamics trajectories generated by a diffusion process, as the collective coordinates emerging from it approximate the eigenfunctions of the Fokker–Planck operator of the process. In ref (127), it has been shown that the diffusion map eigenfunctions are equivalent, up to a constant, to the eigenfunctions of an overdamped Langevin equation.

To compute a diffusion map, one starts by computing a Gaussian kernel
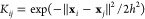
8identical with the one introduced in the previous section. The length scale parameter *h* entering eq 8 has here a specific physical interpretation. It can be seen as the scale within which transitions between two configurations can be considered “direct” or without any significant barrier crossing. In principle, all pairs of configurations enter eq 8, but in practice, only the pairs closer than *h* are relevant in the definition of the kernel *K*_*ij*_, as the contribution of pairs further apart decays exponentially with their distance. For this reason, in practical implementations usually a cutoff distance multiple of *h* is defined and only the distances of pairs within this cutoff are computed.

To provide an approximation of the eigenfunctions of the Fokker–Planck operator, the kernel (eq 8) needs to be properly normalized^128^
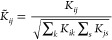
9We note that different normalizations are possible and commonly used to approximate different operators (e.g., graph Laplacian or Laplace–Beltrami instead of Fokker–Planck).^129^ From this normalized kernel, one estimates the diffusion map transition matrix as
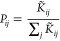
10The resulting elements *P*_*ij*_ can be thought of as the transition probability from data point *i* to data point *j*. Indeed *∑*_*j*_*P*_*ij*_ = 1.

We now consider the spectral decomposition of **P**
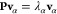
11Since **P** is a stochastic matrix, it is positive-definite, only one of its eigenvalues is equal to 1, and all the others are positive and smaller than 1. If the collection of configurations used to construct the diffusion matrix samples the equilibrium distribution, in the limit of *h* → 0 and infinite sampling, the eigenvectors **v**_α_ converge to the eigenfunctions of the Fokker–Planck operator associated with the dynamics. In practice, for finite *h* and finite sampling, the eigenvectors **v**_α_ provide a discrete approximations to these eigenfunctions, and are called “diffusion coordinates”. Reweighting tricks can be used to apply the diffusion map approach to analyze molecular dynamics trajectories out of equilibrium.^130^

It can be shown that the Euclidean distance in the diffusion coordinates space, scaled by the corresponding eigenvalues, defines a “natural” distance metric on the diffusion manifold, the so-called *diffusion distance*:
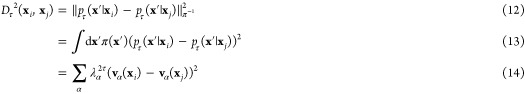
where *p*_*τ*_(**x**^′^|**x**_*i*_) is the probability of being in configuration **x**′ after a time τ for a diffusion process started at position **x**_*i*_, and π is the equilibrium distribution. Because of the equivalence expressed in eq 14, the diffusion coordinates provide an accurate description of the diffusion process, and are usually robust to noise.

Similarly to the other dimensionality reduction algorithms discussed so far, Diffusion map is particularly useful if the spectrum of λ exhibits a gap, say after the *d*-th eigenvalue, with *λ*_*d*__+1_ ≪ *λ*_*d*_. In this case, the sum over the eigenvectors in eq 14 can be truncated including only the first *d* terms and the first *d* diffusion coordinates can be used to characterize the system. The diffusion distance (eq 14) has inspired the definition of a “kinetic distance”,^131,132^ where the same mathematical form is retained but eigenvectors obtained with different spectral methods for the approximation of the dynamics eigenfunctions are used instead.^133,134^

The local scale parameter *h* entering the definition 8 is crucial in determining the transition probability between two configurations. Data configurations coming from the Boltzmann distribution are typically distributed very nonuniformly as they are highly concentrated in metastable states and very sparse in transition states. Therefore, a uniform *h* may be inadequate in MD applications. To address this, the “locally-scaled diffusion map” has been developed by Rohrdanz et al.,^135^ where the parameter *h* becomes position-dependent. The density adaptive diffusion maps approach^136^ is a related technique to deal with highly nonuniform data densities.

Diffusion map has been applied to analyze the slow transitions of molecules,^137,138^ guide enhanced-sampling methods,^139−141^ and have been combined with the kinetic variational principles described in section 6.^142^

#### 3.2.4 Sketch-Map

While building a low dimensional representation of a molecular trajectory, we might be interested in preserving the distances falling within a specific window which is assumed to characterize the important modes of the system under study. This is the main motivation for the introduction of the Sketch-map algorithm.^143^ Sketch-map finds the projection **Y** which minimizes the following loss function:
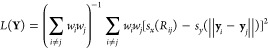
15The above equation differs from the standard MDS loss function (eq 3) in two ways. First, the parameters **w** are introduced to allow to control the importance of the distance between any two points. Second, the distances in the original space and in the projected space are passed through the sigmoid functions *s*_*x*_ and *s*_*y*_ before being compared in the loss. Each sigmoid function depends on the three parameters, σ, *a*, and *b*:
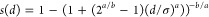
16The parameter σ represents the transition point of the sigmoid (*s*(σ) = 1/2), and it should be chosen as the typical distance that is deemed to be preserved. The parameters *a* and *b* determine the rate at which the functions approach 0 and 1, respectively.

With a careful choice of these parameters, the Sketch-map projection will depend very weakly on distances that are too small or too large, since these will always be squashed either to 0 or to 1, thus giving little or no contribution to the loss of eq 15.

Contrary to the other projection methods described so far, Sketch-map requires solving a highly nonconvex optimization problem (eq 15). In practice, to find a sensible solution, one is forced to use a combination of heuristics,^143^ and in general, there are no guarantees on the computation time needed to find a sufficiently good solution.

The advantage of Sketch-map is that, with a proper selection of the parameters in eq 16, it enables the extraction of relevant structures from a trajectory even when simpler methods fail. However, an adequate choice of parameters can require a lengthy trial-and-error operation, which can be particularly challenging given the absence of guarantees on the time complexity of solving eq 15. Sketch-map has been successfully applied for visualization of free energy surfaces,^143^ biasing of molecular dynamics simulations,^144^ building “atlases” of molecular or materials databases,^145^ and for structure–property prediction.^146^

In the following sections, we describe an approach which allows finding a low dimensional representation of the data working on a very different premise: that relative distances between points are harvested from a stochastic process. This method, like Sketch-map, also works if the data manifold is not isomorphic to a hyperplane.

#### 3.2.5 *t*-Distributed Stochastic Neighbor Embedding

The *t*-distributed stochastic neighbor embedding^147^ (henceforth “t-SNE”) performs dimensionality reduction on high-dimensional data sets following a different route with respect to the approaches discussed so far. The underlying idea (already present in the original Stochastic neighbor embedding (SNE)^148^) is to estimate, from the distances in the high-dimensional space, the probability of each point to be a neighbor of each other point. Then, the algorithm goal is to obtain a set of projected coordinates in which these “neighborhood probabilities” are as similar as possible to the ones in the original space.

The probability that point *j* is a neighbor of *i* is estimated as
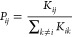
17where *K*_*ij*_ is a Gaussian kernel. In this approach, contrary to what happens in the diffusion map (see eq 10), the length scale parameter *h*_*i*_ is chosen independently for each point *i* by setting
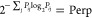
18where Perp is a free parameter called “perplexity”, which roughly represents the number of nearest neighbors whose probabilities are preserved by the projection. Adaptively changing the length scale to match a given perplexity, hence, allows the method to preserve smaller length scales in denser regions of the data set. These probabilities *P*_*ij*_ are then transformed into a joint distribution by symmetrizing the matrix:
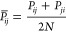
19The neighborhood probabilities in the original space defined in this manner are then transferred to the projected space. For doing this, one needs to choose a parametric form for this probability distribution. At variance with the original SNE implementation, in which a Gaussian form is assumed, in t-SNE, a Student’s *t*-distribution with one degree of freedom is employed:
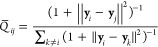
20The shape of this distribution is chosen in such a way that it mitigates the so-called “crowding problem”,^147^ namely the tendency to superimpose points when projecting a high-dimensional data set onto a space of lower dimensionality. Indeed, the heavy tails associated with this distribution allow relaxing the constraints in the distances at the projected space, therefore allowing to a less “crowded” representation.

The two distributions are compared by measuring their Kullback–Leibler (KL) divergence:^150^
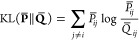
21The feature vectors **y** entering in eq 20 are initialized (randomly in the original formulation, other possible schemes have been proposed^151^) and iteratively modified to minimize the loss function KL(**P̅**||**Q̅**) with a steepest descent algorithm.

The t-SNE loss function eq 21 is nonconvex, making its optimization difficult. In particular, if one uses steepest descent the low-dimensional embedding can differ significantly in runs performed with different initial conditions. This makes t-SNE a method that is, in principle, very powerful and flexible but difficult to use in practice.

Recently, the t-SNE method has been adapted to better fit the needs of molecular simulations. In ref (152), the authors propose a time-lagged version of the method, in the same spirit of TICA (see section 6.1). However, as the authors comment, the time-lagged version distorts the densities from the ones in the original space, which makes the method inappropriate for computing free energies. In ref (153), it is claimed that t-SNE provides a dimensionality reduction which minimizes the information loss and can be used for describing a multimodal free energy surface of a model allosteric protein system (Vivid). The same authors further employ this approach to obtain a kinetic model.^154^

#### 3.2.6 Deep Manifold Learning Methods

Deep learning methods are now frequently used to learn nonlinear low-dimensional manifolds embedding high-dimensional data, and these techniques are also starting to be adopted for analysis and enhanced sampling of molecular simulations.

A popular deep dimensionality reduction method is the autoencoder.^155^ Autoencoders work by mapping input configurations **x** through an encoder network *E* to a lower-dimensional latent space representation **y**, and mapping this back to the original space through a decoder network *D*
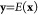
22
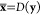
23The network learns a low-dimensional representation **y** by minimizing the error between the original data points **x**_*i*_ and the reconstructed data points **x̅**_*i*_. Note that if *E* and *D* are chosen to be linear maps rather than nonlinear neural networks, then the optimal solution can be found analytically by PCA (section 3.1.1): indeed, the encoder *E* is given by the matrix of selected eigenvectors, and the decoder *D* is given by its transpose.

More involved deep learning approaches to obtain a low-dimensional latent space representation are generative neural networks. Key examples include Variational Autoencoders (VAEs)^156^ and Generative Adversarial Networks (GANs).^157^ VAEs are structurally similar to autoencoders, but involve a sampling step in the latent space that is required in order to draw samples from from the conditional probability distribution of the underdetermined high-dimensional state given the latent space representation, *p*(**x**|**y**). VAEs and other neural network architectures have been widely employed to extract nonlinear reaction coordinates and multidimensional manifolds for molecular simulation.^158−171^ The first goal of these approaches is to aid the understanding of the structural mechanisms associated with rare events or transitions. See section 6 for a deeper discussion of variationally optimal approaches to identify rare-event transitions. Second, the low-dimensional representation of the “essential dynamics” learned by these methods is poised to serve the enhance sampling of events that are rare and particularly important to compute certain observable of interest. This has been approached by constructing biased dynamics in the learned manifold space and reweighting the resulting simulations to obtain equilibrium thermodynamic quantities,^163,165−167^ as well as by selecting starting points for unbiased MD simulations which can then be similarly reweighted to obtain equilibrium dynamical quantities.^161,162,164,172^

GANs implicitly learn a low-dimensional latent space representation in the input of a generator network. Classical GANs are trained by playing a zero-sum game between the generator and a discriminator network, where the generator tries to fool the discriminator with fake samples and the discriminator tries to predict whether its input came from the generator or was a real sample from a database. In the molecular sciences, GANS have (so far) primarily been used for sampling across chemical space; e.g., to aid the optimization of small molecules with respect to certain properties.

For completeness, we mention that there is a third very popular class of generative neural networks called normalizing flows,^173^ which have recently been combined with statistical mechanics in the method of Boltzmann generators,^43^ which facilitate rare-event sampling of molecular systems. However, flows are invertible neural networks and are thereby not inherently performing any dimensionality reduction but rather a variable transformation to a space from which is it easier to sample, but still of the same dimension as the original input. Recently, it has been proposed to combine flows with renormalization group theory to gradually marginalize out some of the dimensions in each neural network layer.^174^ This is a promising direction for combining the tasks of dimensionality reduction and rare-event sampling in the molecular sciences.

## 4 Density Estimation

After the configurations have been projected into a low dimensional space, one can use the representation in this space for estimating the probability density function of the data set. Furthermore, if the representation is of dimension smaller than three, one can directly visualize the probability density ρ. Equivalently, one can also visualize its logarithm which, if the simulation is performed in the canonical ensemble at temperature *T*, is equal to the free energy; that is, *F*(**y**) = −*k*_B_*T* log(ρ(**y**)).

Density estimation is of great interest well beyond molecular simulations, and is a key working tool in unsupervised machine learning. Density estimation consists of estimating the underlying probability density function (PDF) from which a given data set has been drawn. Although its main applications relate to data visualization (as in the case of visualizing the free energy surface), it is also part of the pipeline of other analysis methods such as kernel regression,^175^ anomaly detection,^176^ and clustering (see Section 5).

The natural connection between density estimation and free energy reconstruction can be exploited to increase the efficiency of simulation analyses. For example, many kinetic analysis methods rely on density estimation^177−181^ (see Section 6).

In the case of equilibrium molecular systems, the PDF as a function of a feature vector **y** is in principle known exactly. In the canonical ensemble, this is given by,
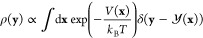
24where  is the function which enables the computation of the feature vector **y** as a function of the coordinates **x**. In practice, however, calculating this integral is not possible, and the resulting PDF can only be estimated approximately following one of the procedures described in this section.

**Figure 5:**
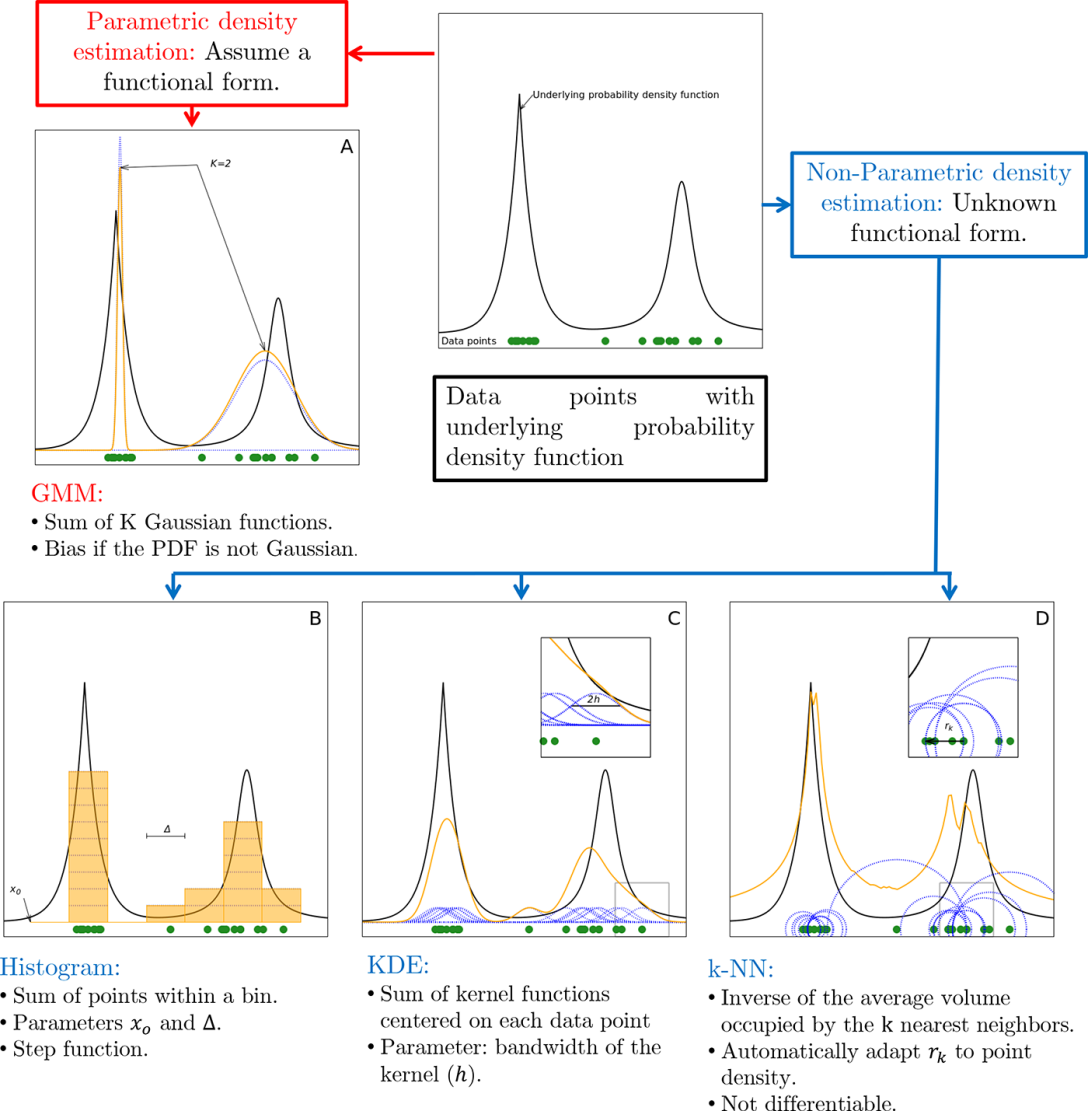
Graphical summary of the density estimation methods. Different density estimates on 20 1D data points (green) extracted from a mixture of a Laplace and a Cauchy distribution (black line).

Density estimation methods can be grouped into two categories, namely, parametric and nonparametric methods (see Figure 5). In the first one, a specific functional form for the PDF is chosen and its free parameters are inferred from the data. In contrast, nonparametric methods attempt to describe the PDF without making a strong assumption about its form.^182^

The choice between parametric and nonparametric methods is heavily problem dependent. As a general rule, when the origin of the data permits a reasonable hypothesis for the functional form of the PDF, parametric density estimation should be preferred, since it quickly converges with relatively few data points. If the functional form is unknown, however, it is often better to sacrifice performance than to risk introducing bias into the estimation.

### 4.1 Parametric Density Estimation

In parametric density estimation a fixed functional form of the PDF is assumed and one estimates its parameters from the data. For example, if one assumes that the data are sampled from a Gaussian distribution one can then estimate the density by simply computing mean and variance of the data. Such a procedure is common throughout various scientific branches. However, this procedure can lead to an error in the estimate that cannot be reduced by increasing the number of observations, since the error can be brought to zero only if the data points are truly generated from a Gaussian distribution. To add flexibility to this approach and, therefore, alleviate the bias inherent in assuming the form of the distribution a priori, a common technique is to model the PDF as a mixture of *K* distributions
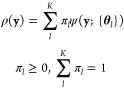
25where ψ(**y**;{**θ**_*l*_}) is a function that depends upon the set of parameters **θ**_*l*_ and *π*_*l*_ is the weight of this function in the estimate. A common choice for ψ is a Gaussian, leading to the Gaussian mixture model (GMMs).^183^

The parameters {**θ**_*l*,_π_*l*_} can be estimated by a maximum likelihood method, namely
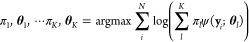
26where the sum over *i* runs over the *N* observations **y**_*i*_, *i* = 1, ..., *N*. This likelihood function can then be optimized through (for example) an expectation-maximization scheme.^183^

A general problem of GMMs is that, since the likelihood to be optimized is a nonlinear function of the parameters, finding its global maximum is typically very difficult. Another critical issue in this approach is the choice of the hyperparameter *K*, that is, the number of functions used in the mixture. There is no straightforward relationship between the quality of a density estimate and *K* because the best choice strongly depends on the shape of the PDF. The choice of *K* is a model selection problem,^184^ which can be approached by maximizing the likelihood on a validation data set.^185^ Alternative Bayesian approaches for this problem involve maximizing the marginal likelihood with respect to *K* or using a Dirichlet process prior distribution for the hyperparameter *K*.^186^

There are numerous applications of mixture models in the analysis of molecular simulation. In refs.,^187,188^ the free energy surfaces of biomolecules are reconstructed as a sum of Gaussian functions. In refs.,^189,190^ GMMs are employed as a basis for an enhanced sampling algorithm in a way that follows the spirit of metadynamics^191^ but in which the bias potential is updated to be the sum of few Gaussian functions. In this case, the number of basis functions can be adjusted by using variationally enhanced sampling.^192^ Moreover, in ref.,^181^ GMMs are used as basis for the MD propagator in a kinetic model (see section 6), while in ref (193), they are used for atomic position reconstruction from coarse-grained models.

### 4.2 Nonparametric Density Estimation

#### 4.2.1 Histograms

Nonparametric methods avoid making strong assumptions on the functional form of the PDF underlying the data. The most popular nonparametric method, especially in the molecular simulation community, is the histogram.^194−198^

In this method, the space of the data is divided into bins and the PDF is estimated by counting the number of data points within each bin. In one dimension, denoting the center of bin *I* as *y*_I_, and taking *y*_I_ = *y*_0_ + *I*Δ, where Δ is the bin width, we have
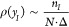
27where *n*_*I*_ is the number of configurations within bin *I*, and can be computed as , where χ_[*a*,*b*]_(*y*) = 1 if *y* ∈ [*a*, *b*] and 0 otherwise.

Under the assumption that the different observations are independent, *n*_*I*_ is sampled from a binomial distribution *n*_*I*_ ∼ *B*(*N*, *p*_*I*_) where (in a one-dimensional feature space)  is the probability of observing a configuration in the bin. The expected value of *n*_*I*_/*N* is equal to *p*_*I*_, which implies that the estimator in eq 27 is correct only if  can be approximated with Δ·ρ(*y*_*I*_). If Δ is too large, this approximation is not valid and the density estimation becomes too coarse-grained, thereby inducing a systematic error, which is referred to as *bias*.

The variance of *n*_*I*_ is Var(*n*_*I*_) = *N p*_*I*_ (1 – *p*_*I*_), and the statistical error on the density estimate in eq 27 can be estimated as
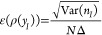
28
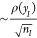
29where to go from the first to the second line we used the fact that *p*_*I*_ ≪ 1 and *p*_*I*_ ≅ ρ(*y*_*I*_)Δ. If one chooses a value of Δ that is too small, the number of configurations within the bin *n*_*I*_ becomes small, and the resulting error, commonly referred to as *variance*, becomes large. In practice, the value of Δ can be chosen by considering the so-called bias/variance trade-off in which both small variance and small bias are desired, but decreasing one often increases the other.^182^ This trade-off is common to all nonparametric density estimators.

Histograms are typically used to estimate the density for *d* ≤ 3 since in higher dimensions the estimator becomes noisy since an increasing number of bins will be either empty or only visited a few times. This problem can be alleviated only if one exponentially increases the number of data points with *d*: a manifestation of the curse of dimensionality.^199^ Finally, another drawback of the histogram is that its estimate of the PDF is not differentiable.

#### 4.2.2 Kernel Density Estimation

Kernel density estimation (KDE) partially overcomes the problems associated with histogramming. KDE approximates the PDF of a data set as a sum of kernel functions centered at each data point. In one dimension, the approximation reads
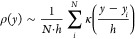
30where the kernel function κ is chosen as a unimodal probability density symmetric around 0. Particularly, it satisfies the properties
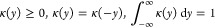
31The estimator depends on the kernel function of choice and the hyperparameter *h* > 0 (i.e., the bandwidth). Some popular examples of kernels are summarized in Table 1.

**Table 1:** Some Widely Used Kernels for Density Estimation

uniform	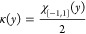
triangle	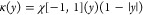
Epanechnikov	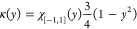
cosine	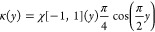
Gaussian	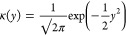

The Gaussian kernel is arguably the most used. The Epanechnikov kernel is optimal in the sense that it minimizes the mean integrated squared error (MISE).^200^ However, experience has shown that the impact of the choice of the kernel on the quality of the estimation is lower than the choice of the bandwidth.^201^

The bandwidth hyperparameter *h* plays a role similar to the bin width parameter Δ in the histogram method. *h* is also referred to as the “smoothing” parameter; since the larger it is, the smoother the resulting PDF estimate is. However, smoothing too much can lead to artificially delete important features on the PDF. The dependence of the MISE on *h* can be decomposed into two terms, namely, the *bias*, which scales as *h*^2^, and the *variance*, which corresponds to the error induced by the statistical fluctuations in the sampling and scales as .^202^

As in the case of the histogram, the optimal value of *h* should be chosen as a trade-off between the bias and the variance terms. Much research has been focused on the optimal choice of *h* in eq 30.^203−205^

Alternatively, the bandwidth selection problem can be addressed by introducing an adaptive kernel with a smoothing parameter that varies for different data points.^206,207^ A position-dependent bandwidth can be obtained by optimizing a global measure of discrepancy of the density estimation from the true density.^182,208^ However, this global measure is typically a complex nonlinear function,^209^ and its optimization can be prohibitive for large data sets. A more feasible approach, first proposed by Lepskii,^210^ is to adapt the bandwidth to the data locally. This approach has been further developed by Spokoiny, Polzehl, and others.^211−216^

Kernel density estimation can also be used in more than one dimension by employing multivariate kernels. However, the number of hyperparameters increases with the number of dimensions. In the case of the multivariate Gaussian kernel, the parameters can be summarized in a d×d symmetric matrix **H**, which plays a role analogous to *h* in the one-dimensional case. The corresponding estimator is
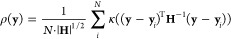
32In comparison with histograms, kernel density estimation has the advantage that it is differentiable. KDE is gaining increasing popularity in the analysis of molecular dynamics simulations, leading even to the development of specific parameters tailored for MD.^217^ In addition to their use in visualization of free energy surfaces^218^ and the construction of kinetic models,^178^ KDEs have been applied in the study of nonexponential and multidimensional kinetics^219^ and the computation of entropy differences.^220^

#### 4.2.3 *k*-Nearest Neighbor Estimator

Another route for estimating the density of a data set is the *k*-nearest neighbor (*k*-NN) estimator.^221^ In this method, the probability density *ρ*_*i*_ = ρ(**y**_*i*_) is estimated as
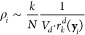
33where *V*_*d*_ is the volume of the unitary sphere in dimension *d* and *r*_*k*_(**y**_*i*_) is the distance between **y**_*i*_ and its *k*-th nearest neighbor point.

The *k*-NN method can be thought of as a special kernel density estimation with local bandwidth selection, where the role played by *k* is similar to that of *h* in the KDE. The statistical error induced of this estimator is given by^13^
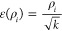
34The higher the value of *k*, the smaller the variance of the estimator, but the larger the bias, since this error estimate is valid only if the probability density is constant in the hypersphere of radius *r*_*k*_(**y**_*i*_) centered on **y**_*i*_. The *k*-NN method can in principle be used for estimating the PDF in any dimension *d*. However, if *d* = 1, the PDF estimated in this manner cannot be normalized since the integral for the whole space of 1/*r* does not converge.^222^

The *k*-NN method is also affected by the curse of dimensionality. Indeed, it can be shown that for any fixed number of data points, the difference between the distance from the *k* nearest neighbor (*r*_*k*_) and the distance from the next nearest neighbor (*r*_*k*__+1_) trends to 0 when *d* → *∞*,^223^ leading to problems in the definition of the density. Much effort has been put into bypassing this limitation.^224^ One approach involves avoiding estimating the density in the full feature space and instead estimating it in the manifold that contains the data (which usually has a much lower dimensionality). This approach, first suggested in ref (225), was further developed in ref (13) with a specific focus on the analysis of MD trajectories.

While in molecular systems the configurations are defined by a high-dimensional feature space, restraints induced by the chemical and physical nature of the atoms prevent the system from moving in many directions (for example, in the direction which significantly shortens a covalent bond). In practice, these restrains reduce the dimension of the space to a value which is referred to as an *intrinsic dimension*. The intrinsic dimension can be estimated, for example, using the approaches in refs (226−229). The probability density can then be estimated using eq 33, where the dimension of the feature space *d* is replaced with the intrinsic dimension, which can be orders of magnitude smaller, making the estimate better behaved. This framework also addresses the problem of finding a *k* that yields sufficiently small variance and bias. Ref (13) proposes that the largest possible *k* for which the probability density can be considered constant (within a given level of confidence) for each data point separately. This optimal *k*, which is point-dependent and denoted by *k*_*i*_, is then used to estimate the probability density by a likelihood optimization procedure allowing for density variation up to a first-order correction. The estimator of the density then becomes
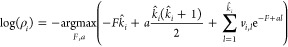
35where *v*_*i*__,*l*_ = *V*_*ID*_ (*r*_*l*_^*ID*^(**y**_***i***_) – *r*_*l–*1_^*ID*^(**y**_***i***_)) is the volume of the hyperspherical shell enclosed between the *l*th and the (*l* – 1)th neighbor of the configuration **y**_*i*_. This approach enables the estimation of the probability density directly in the space of the coordinates introduced in section 2 (for example, the dihedral angles) rather than performing a dimensionality reduction with one of the methods described in section 3 in advance or by explicitly defining a collective variable for describing the system.

In the field of MD simulations, *k*-NN has been used as part of the pipeline for more complex analyses^230,231^ and for computing both entropies^232,233^ and free energies.^234,235^

## 5 Clustering

Clustering is a general-purpose data analysis technique in which data points are grouped together based on their similarity according to a suitable measure (for example, a metric). In molecular simulations, the use of clustering is very common, since clusters can be seen as a way of compactly representing a complex multidimensional probability distribution. Clustering, thus, performs an effective dimensionality reduction in a manner which can be seen as complementary to the approaches described in section 3. It is well-known that there is no problem-agnostic measure of the success or appropriateness of a given clustering algorithm or its results.^236^ Thus, it is crucial to choose a clustering algorithm based on what is known about the data set and what is expected about the result. In particular, in the field of molecular simulations, we can conveniently differentiate the following two classes of techniques.

**Figure 6:**
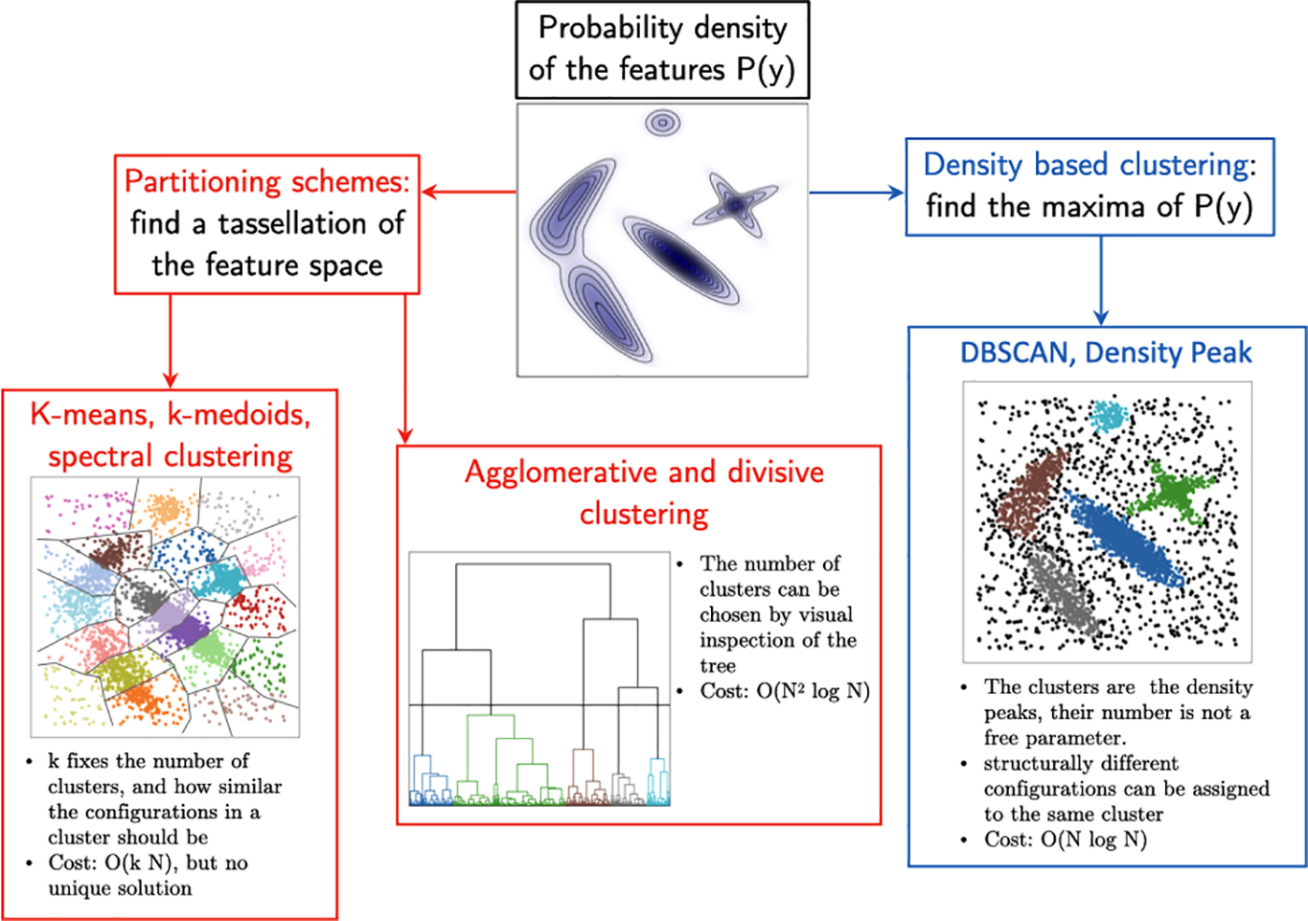
Cartoon illustration of the different clustering schemes.

- **Partitioning schemes:** In these approaches, the clusters are groups of configurations which are similar to each other and different from configurations belonging to other clusters. These clusters define a partition (or tessellation) of the space in which the configurations are defined.
- **Density-based schemes:** In these approaches, the clusters correspond to the peaks of the probability distribution from which the data are harvested (or, equivalently, to the free energy minima). Data points belonging to different clusters are not necessarily far apart if they are separated by a region where the probability density is low (see Figure 6).

The most striking difference between these two distinct approaches emerges if one attempts to cluster a set of data harvested from a uniform probability distribution. Using a partitioning scheme, one can find any number of clusters depending on the chosen level of resolution. On the other hand, using a density based scheme, one will obtain a single cluster. Clearly, which approach one should employ strongly depends upon the purpose of the analysis. For instance, if one wants to find directly the metastable states from a cluster analysis of the system, one should use a density-based clustering approach. If, instead, one wants to find an appropriate basis to represent the dynamics, as in kinetic modeling methods (see section 6), a partitioning scheme may be more appropriate, since these schemes allow one to control how similar the configuration assigned to the same cluster are.

### 5.1 Partitioning Schemes

Partition-based algorithms aim to classify the configurations in sets (i.e., clusters) that include only similar configurations. These algorithm can be further divided into two classes.

- **Centroid-based/Voronoi tesselation algorithms**: In these methods, the number of clusters is determined by a specific parameter, which can be a cutoff specifying the maximum allowed distance between two configurations assigned to the same cluster or, alternatively, the number of clusters. The well-known *k*-means and *k*-medoids algorithms belong to this class, as well as the faster but less optimal *leader algorithms k*-centers and regular-space clustering. In all of these methods, each cluster is associated with a so-called centroid, which is a configuration representing the content of the cluster. This centroid induces a so-called *Voronoi*-tesselation, which divides space such that each point is associated with the nearest centroid, in the chosen distance metric.
- **Hierarchical/agglomerative and divisive clustering**: Here, the choice of the number of clusters is deferred, being possible to run the algorithm without setting it in advance. A (usually binary) tree is constructed according to a linkage criterion, and the number of clusters can be selected by appropriately “cutting” the tree, usually by visual inspection and taking into account the scope of the analysis. These approaches are commonly referred to as hierarchical clustering, which can be “agglomerative” or “divisive” depending on whether the data points are first considered to be individual clusters or members of a single cluster, respectively.

#### 5.1.1 *k*-Means and *k*-Medoids

Arguably, the most popular clustering schemes in molecular simulations and beyond are the centroid-based methods *k*-means^237^ and *k*-medoids.^238^ In both cases, the number of clusters *k* is chosen before performing the algorithm.

In *k*-means, also known as Lloyd’s algorithm,^237^ the cluster centers are *k* configurations whose position **x**_*c*,*i*_, *i* = 1, ..., *k* correspond to the *mean* of the coordinates (or the features) of all the cluster members. For a given choice of *k* cluster centers, the quality of the clustering is defined by a loss *L*(**x**_*c*_). This loss is defined as the sum of the square of the distances from each configuration in the data set to the cluster center to which the configuration is assigned. Therefore, the *k*-means clustering problem is formulated as an optimization problem in which the best solution (i.e., set of cluster centers) is the minimum of *L*(**x**_*c*_) with respect to **x**_*c*_. This optimization is known to be NP-hard.^239^ To find an approximate solution, ref.^237^ proposes the following iterative procedure:

1. *k* members of the data set are randomly chosen as centers.
2. Each configuration in the data set is assigned to its closest center.
3. The centers are recomputed according to the new assignments.
4. Points 2 and 3 are repeated until the cluster membership does not change.

Because of the glassy nature of *L*(**x**_*c*_), the outcome of the algorithm is heavily dependent on the initialization step. Thus, it is often necessary to repeat the procedure above with different initial centers to obtain reasonable results. In light of this drawback, initialization schemes have been proposed that improve the tractability of the problem by speeding up convergence.^240^ A “minibatch” *k*-means version has also been proposed, which alleviates the problem of trapping in local minima and it is faster than the original method (in which all the configurations are simultaneously used in the optimization).^241^ In a closely related approach, called fuzzy *c*-means,^242,243^ each point does not belong to a single cluster, but rather its degree of membership to all possible clusters is represented by a vector **u** with *k* components such that Σ_*i*=1_^*k*^*u*_*i*_ = 1. *k*-means scales as  in the number of configurations *N* and the number of iterations *i*. The number of iterations *i* needed to achieve convergence depends on how the data is distributed and will often depend on *N*, but in practice, *k*-means is often terminated when a fixed number of iterations has been reached.

In *k*-means, the cluster centers in the first step are configurations in the data set, but in the following steps their positions are adjusted, and (thus), they will no longer correspond to configurations in the original data set. This means that *k*-means is applicable if an explicit feature representation is given, but not if only a distance metric is defined (e.g., when a pairwise RMSD minimal distance is used). The *k*-medoids algorithm^238^ ensures that each cluster centroid always corresponds to a configuration in the data set, offering an alternative. In this approach, each cluster center in a given iteration is chosen as the configuration *in the original data set* which minimizes the sum of the distances from the cluster members. A minibatch strategy can also be employed in *k*-medoids. With sufficient data, *k*-means and *k*-medoids are expected to give qualitatively similar results. The key advantage of the latter is interpretability; for example, in a molecular simulation application each *k*-medoids cluster center can be easily visualized as a configuration present in the raw data set. Additional advantages of *k*-medoids are that it is less sensitive to the presence of outliers and that it can be used with a distance measure between pairs of coordinates other than Euclidean distance. The *k*-medoids algorithms scales as , and it is hence difficult to use for large data sets.^244^ In both *k*-means and *k*-medoids, the choice of *k* is an open problem. A common practice consists in running the algorithm with increasing *k* values, and then plot the loss as a function of *k* (the so-called scree plot^245^) looking for an elbow. Other validation indexes, like the Silhouette^246^ can be also employed to this end.

#### 5.1.2 Leader Algorithms: *k*-Centers and Regular-Space Clustering

As *k*-means and *k*-medoids may require many passes through the data set, they can become extremely expensive for large data sets. In his book,^247^ John Hartigan proposed a *leader algorithm* in which it is necessary to iterate over the data only twice: the first time to assign *k* centroids and the second time to assign all data points to centroids. This results in a fast runtime of  for *N* configurations. The most popular algorithm based on this idea is *k*-centers.^248^ The objective of *k*-centers is to maximize the distance between cluster centers. To employ the algorithm, the first cluster center is chosen randomly from the data set. Then, the next cluster center is chosen as the configuration in the data set that is farthest from the previously chosen configuration according to a distance measure. This procedure is iterated until the desired number of centers have been obtained. In the second pass, each configuration in the data set is assigned to the cluster defined by the closest centroid. *k*-centers scales as  for *N* configurations.^248^ In the absence of “ties”, *k*-centers is deterministic after the selection of the initial cluster center, but it depends on the order in which the data points are traversed, which may be arbitrary in many applications. For large data sets, the results are approximately independent of these choices. It is important to consider whether the *k*-centers objective is appropriate for the application at hand, since by definition it will choose outlying configurations as cluster centers,^38^ and this may not be desired. Subsampling the data^20^ or using a hybrid clustering scheme^249^ may mitigate this effect.

Another variant of the leader algorithm, which is equally fast but less sensitive to outliers is *regular space clustering*.^21^ Here, instead of fixing the number of clusters, one fixes a minimal distance cutoff *d*_*c*_. The first cluster center is chosen at random. Then one cycles through the data set, accepting a new data point as a cluster center when its distance to all existing cluster centers is greater than *d*_*c*_.

Another similar partitioning procedure was introduced by Daura et al.^250^ In this approach, one also chooses a cutoff distance *d*_*c*_, under the assumption that configurations which are closer than *d*_*c*_ are similar enough to be assigned to the same cluster. For each configuration *i*, one then estimates the number *n*_*i*_ of other configurations within *d*_*c*_. The first cluster center is the configuration with the highest *n*_*i*_; namely, with more neighbors within a distance *d*_*c*_. All the configurations within *d*_*c*_ from the first center form the first cluster. The second cluster center is then the configuration with the highest *n*_*i*_ after excluding the configurations are already assigned to the first cluster. The second cluster is formed by all the configurations within *d*_*c*_ from the second center that do not belong to the first cluster. This procedure is iterated until all the configurations are assigned to a cluster. Its computational cost scales as  for *N* configurations, which can be reduced to  by using a smart neighbor search algorithm. This procedure is deterministic: given a set of configurations and a cutoff distance *d*_*c*_ it produces a unique clustering partition, except if for two data points the number of neighbors *n*_*i*_ is equal.

Since (like *k*-medoids) leader algorithms use available data points as centroids, they are compatible with clustering scenarios, where only a pairwise distance metric is given but no explicit feature representation. In early developments of kinetic models for molecular simulation data (see section 6); when pairwise RMSD was used, *k*-centers and regular space clustering were frequently employed for clustering steps of kinetic modeling algorithms.^20,21,251−254^ Other—and especially more recent—applications are mostly based on *k*-means or its minibatch variant^29,40,255−259^ and, less frequently, *k*-medoids^260^).

#### 5.1.3 Spectral clustering

One of the drawbacks of the approaches described in the previous sections is that they all rely on the analysis of the distances between pairs of configurations. Such distances, if computed using all the coordinates, can be affected by the noise induced by the high dimensionality. Moreover, the need to deal with distances does not allow the use of other similarity measures that, for instance, do not respect the triangular inequality.

Spectral clustering addresses the problem of clustering by an approach that does not require computing distances. It uses a set of pairwise similarities to define a weighted undirected graph, in which each data point corresponds to a vertex and the edges connecting two vertices *i* and *j* are associated with a weight *W*_*ij*_. For convenience, one can organize these weights in a matrix **W** and define the diagonal degree matrix **D** as *D*_*ii*_ = *∑*_*j*_*W*_*ij*_, and the graph Laplacian matrix as **L** = **D** – **W**.

The transformation of the pairwise similarities into a graph can be done in three ways: (1) by connecting all points whose similarities are equal or greater than a given threshold, (2) by connecting each point to the *k* points which are most similar to it, and (3) by treating the similarities directly as weighted edges of a fully connected graphs.

Once the graph is built, the method attempts to divide it into clusters in such a way that the edges of the graphs associated with data points belonging to different clusters have small weights, and the edges within a cluster have large weights. One then defines the cost associated with a given partition into *k* clusters as
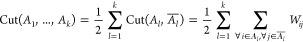
36where Cut(*A*_*l*_, *A̅*_*l*_) defines the cost associated with dividing the graph into a set *A*_*l*_ and its complement *A̅*_*l*_ (the elements not belonging to *A*_*l*_). The direct application of this principle is referred to as the “min-cut” approach,^261^ which is found empirically to produce imbalanced partitions^262^ since in many cases generates clusters with a single element. This problem can be addressed by redefining the cost function taking into account the size of the clusters. This gives rise to a BalancedCut cost function
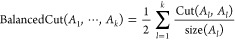
37Two successful ways exist to define the size of the clusters within the BalancedCut objective function. In the RatioCut approach,^263^ the size of a cluster size(*A*_*l*_) is simply measured by the number of vertices in the cluster, while in the normalized cut (Ncut) approach^264^ the size of the cluster is measured by the cumulative connectivity of all data points belonging to the cluster size(*A*_*l*_) = ∑_∀*i*∈*Al*_*D*_*ii*_.

To find the cluster partition that minimizes this quantity one defines **H** as a *N* × *K* indicator matrix, where *H*_*il*_ has a discrete positive value if the element *i* belongs to the clusters *l* and zero otherwise. It can be shown that minimizing the balanced cut can be reformulated as a minimization the trace of the matrix **H**^*T*^**HL** with the constraint **H**^*T*^**H** = **I** (in the *RatioCut* case) or **H**^*T*^**DH** = **I** (in the *Ncut* case). The latter case can be rewritten as the minimization of the trace of **H̃**^T^**L̃H̃** with the constraining **H̃**^T^**H̃** = **I**, by defining a normalized indicator function as  and a normalized Laplacian as . Unfortunately, both problems are NP-hard because of the discreteness of the values of **H**. In spectral clustering, this condition is relaxed, allowing **H** to take arbitrary real values. In this manner the minimization of the trace can be handled as an eigenproblem, and the trace is minimized by a matrix **H** composed of the first *k* eigenvectors of **L**. However, the relaxation of the discreteness condition implies that the columns of **H** do not provide indicator functions but a continuous vector space. Therefore, the final step of a spectral clustering algorithm involves clustering the configurations within the **H** space using a *k*-means algorithm (see section 5.1.1).

To summarize, the spectral clustering algorithm consists of (1) building the weighted graph from the similarities/distance matrix, (2) computing the (normalized) graph Laplacian and obtain its first *k* eigenvectors, and (3) using these eigenvectors as input for a *k*-means clustering step. The value of *k* can be chosen with the same criteria described in section 3.1.1; namely, if a gap in the eigenvalue spectrum is present, one should choose the value of *k* that preserves eigenvectors corresponding to eigenvalues above this gap.

Apart from the graph-based interpretation illustrated in this section, the algorithm has been derived in other frameworks and has many variants, mostly differing in the way in which the graph is generated or the way in which the Laplacian is normalized. In a specific formulation,^265^ spectral clustering can be seen as a Kernel PCA (described in section 3.2.2), followed by a *k*-means clustering step; under certain conditions, it has been shown to be equivalent to kernel *k*-means^266^ The interested reader is encouraged to check specific reviews on spectral clustering^262,267^ for more details on the various existing approaches.

This clustering procedure is powerful and robust, and it has been widely applied for analyzing molecular dynamics trajectories.^268−270^ The graph can be generated, for example, using the RMSD between structures as a similarity measure combined with a squared exponential kernel.^268^ However, spectral clustering techniques differ in many details: the way of generating the graph, the ways of normalizing the Laplacian, the way of choosing *k* in the absence of a clear gap. Some recommendations and rules-of-thumb can be found in ref (267), but a careful system-dependent evaluation is typically necessary.

#### 5.1.4 Hierarchical Clustering

A significant drawback of *k*-means and related algorithms is the necessity of choosing *k* a priori. In hierarchical clustering, this problem is circumvented by building a tree structure, called a dendrogram, which represents an ensemble of clustering models with every possible *k*. One can then choose the most appropriate partition a posteriori from the dendrogram. Hierarchical clustering approaches require defining a dissimilarity function and a linkage criterion (the latter is sometimes referred to as an objective function). The dissimilarity function does not need to be a metric. In particular, it does not need to satisfy the triangular inequality, which yields great flexibility.

Hierarchical clustering algorithms can be classified into two categories; namely, agglomerative and divisive algorithms. In agglomerative clustering, each configuration is initially assigned to a different cluster. At every iteration two existing clusters are combined, so the next level of the dendrogram has one fewer cluster. In divisive clustering, the data set starts as a single cluster of all *N* configurations, and at each iteration an existing cluster is split into two. Divisive clustering can be computationally intractable because the number of possible divisions scales unfavorably as clustering proceeds; therefore, we restrict the remainder of this overview to agglomerative clustering only (the reader is referred to ref (238) for a detailed discussion of this topic).

Agglomerative clustering is initialized by considering every configuration as a different cluster to create *N* singleton clusters. Then, the closest two configurations are combined, leaving *N* – 1 clusters. Which pair of clusters is linked in each step of the algorithm is decided by a *linkage criterion*, which is different in different algorithms. In single linkage^271^ at a given step of the algorithm one links together the two clusters *A* and *B*, which are closer according to the dissimilarity measure min(*d*(*a*, *b*)) for *a* ∈ *A*, *b* ∈ *B*. In complete linkage,^272^ the dissimilarity measure used to decide which clusters are linked is max(*d*(*a*, *b*)) for *a* ∈ *A*, *b* ∈ *B*, and in average linkage^273^ is the average value of the distance between the elements of the two clusters. Finally, in Ward linkage,^274^ one agglomerates the pair of clusters that leads to minimum increase of the variance of *d*(*a*, *b*) estimated for the data belonging to the cluster created upon agglomeration. Agglomeration continues until only a single cluster remains.

The dendrogram comprising the clustering model can then be “cut” for any number of clusters 2 ≤ *k* ≤ *n*. The change in the value of the linkage criterion between levels of the dendrogram can be used to inform where it should be cut^275^ (cutting the dendrogram before the sharpest increase is called the “elbow method”). It is clear that the choice of linkage criterion will significantly impact the result; for example, for randomly distributed data in two dimensions, single linkage will create snake-like clusters and complete linkage will create circle-like clusters.^276^

In molecular kinetics (see section 6), agglomerative clustering is less frequently employed than partitioning schemes, such as *k*-means, since the computation of every pairwise dissimilarity scales as *n*.^2^ When agglomerative clustering has been used to build kinetic models, for example, the simulation data to be clustered was first significantly subsampled to accommodate this scaling.^253,277^ It was shown in ref (278) in the context of kinetic modeling of molecular simulations (see section 6.3) that agglomerative clustering with Ward’s linkage produces similarly accurate results to clustering with *k*-means; indeed, the Ward objective function can be linked to that of *k*-means.^279^

### 5.2 Density-Based Clustering

In molecular dynamics simulations, the configurations are harvested from a probability distribution. This distribution is often characterized by the presence of relatively isolated probability peaks that typically correspond to metastable states of the system. Density-based clustering algorithms can be used to find these peaks directly. In these approaches, one first estimates the density of each configuration using one of the approaches described in section 4. Then, one looks for the peaks in this density—these peaks define the clusters. Since a probability peak can in principle have any shape (for example, it can be strongly elongated in one direction), the configurations assigned to a cluster are not necessarily similar. Different density-based clustering algorithms differ in their strategies for finding the density peaks.

Possibly the oldest and most famous algorithm for finding the peaks of a density in feature space is the mean-shift approach.^280^ The idea at the basis of this algorithm is simple: For each data point, follow the gradient of the density in ascending direction until you arrive at a local maximum. All the points arriving at the same maximum define then a cluster. Thus, starting from a feature vector *y*, one first estimates the direction of the gradient of the density as
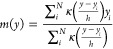
38where  is a kernel function, satisfying the conditions in eq 31. If the kernel bandwidth *h* is small and the data points are many, *m* points in the direction of increasing ρ. Therefore, one can update the feature vector *y* to *y* + *m*(*y*), and iterate the procedure until *y* does non change significantly anymore. This approach, very popular in image analysis, has been also applied to the analysis of molecular dynamics trajectories,^281,282^ but it is rather computationally expensive since one is forced to run the iterative procedure described above for each point in the data set. Moreover, the approach inherits the problems of kernel density estimation. If the bandwidth *h* is too small, the algorithm tends to converge to spurious maxima, whose existence is only due to undersampling. If, instead, *h* is too large, some relevant maxima can be missed due to excessive smoothing. The approaches described in the following sections partially overcome these problems and are, in particular, less sensitive to noise and more computationally efficient.

#### 5.2.1 DBSCAN

Density-based spatial clustering of applications with noise (DBSCAN)^283^ defines clusters as connected regions with density above a threshold surrounded by regions with a density below this threshold. In the original formulation, the density threshold is defined by two parameters: a neighborhood distance (*h*) and the minimum number of configurations within this distance needed for considering a given configuration above the density threshold (MinPts).

In practice, one first estimates the density by counting how many configurations are within the neighborhood defined by *h* for each configuration *i*. Note that in this method the densities estimated at a given configuration are proportional to those estimated using a uniform kernel with *h* = ϵ (see eq 30). Then, the configurations whose number of neighbors within ϵ is greater than or equal to *MinPts* are considered above the threshold and called *core points*. A configuration *j* is said to be “directly reachable” from the configuration *i* if *i* is a core point and *j* is within ϵ of *i*. A point *j* is “reachable” from *i* if there is a sequence of points *i*_1_, ..., *i*_*n*_ with *i* = *i*_1_ and *j* = *i*_*n*_ in which each *i*_*l*__+1_ is directly reachable from *i*_*l*_ for all *l*. Two configurations *i* and *j* are called “density-connected” if there is a configuration *k* such that both *i* and *j* are reachable from *k*. It is important to note that while the reachability property is not symmetric (e.g., configuration *j* can be reachable from configuration *i* without *i* being reachable from *j*), the density connection is a symmetric property. Using these definitions, a cluster is defined as a set of configurations that are all density-connected. Configurations that are not core points nor density connected are classified as *noise points*.

Since it is a density based method, DBSCAN does not require one to specify the number of clusters in the data a priori, in contrast to most of the partitioning schemes. Moreover, DBSCAN can find arbitrarily shaped clusters. However, the choice of the proper combination of parameters MinPts and ϵ can be difficult when the densities across configurations are not uniform or when hierarchical structures are present.^284^ To solve these issues, several variants have been proposed (see ref.^285^ for a recent survey). Of particular relevance are OPTICS^286^ and HDBSCAN,^287,288^ both of which provide a hierarchical view of the cluster structure.

DBSCAN had been employed to find representative structures from MD simulations,^289,290^ as well as to find regions characterized by different molecular densities,^291,292^ in this case using molecules as data points. HDBSCAN has recently gained attention also in the analysis of MD trajectories,^293−296^ mostly due to its capability to reveal hierarchical structures.

#### 5.2.2 Density Peak Clustering

Density peak clustering^297^ finds the density peaks according to a different procedure than DBSCAN. Density peak clustering is based on the idea that if a configuration is close to a local maximum of the probability density, then it is surrounded by neighbors with lower density—or, equivalently, it is likely to be at a relatively large distance from any configurations with a higher density.

These simple qualitative criteria are implemented as follows. For each configuration, one first estimates the density *ρ*_*i*_ of configuration *i* (using any of the approaches described in section 4). Then, one computes the minimum distance between the configuration *i* and any other configuration with higher density
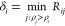
39where *R*_*ij*_ is the distance between configurations *i* and *j*. According to eq 39, the value of *δ*_*i*_ of the point with highest density remains undefined, so it is assigned to a value higher than the rest by convention. Cluster centers are identified as configurations for which the value of *δ*_*i*_ and *ρ*_*i*_ are both anomalously large. This is because a center is expected to have both a high density and a large distance from configurations with higher density.

To select the centers in practice, it is proposed to plot the value of *δ*_*i*_ as a function of *ρ*_*i*_ for each configuration. In this visualization, the configurations corresponding to density peaks emerge as outliers and can be recognized by the user in an interactive way.

Depending on the application, this interactive step may not be feasible; in this case, an automatic criterion is needed. However, defining a quantitative criterion for automatically choosing the centers according to this qualitative definition is nontrivial. In the original implementation^297^ it is proposed to find the number of clusters by a criterion similar to that used in spectral clustering and PCA; namely, one considers the values of *γ*_*i*_ = *δ*_*i*_*ρ*_*i*_ sorted in descending order. If a gap is present (say, before *γ*_*k*_), set the cluster centers to be all configurations with *γ*_*i*_ > *γ*_*k*_.

Once the cluster centers are determined, the rest of configurations are assigned to the same cluster as their nearest neighbor with higher density.

The original procedure^297^ has been successively improved and modified in order to address some of its pitfalls. Faster versions have been generated, both improving the quality of the implementation^298^ and making use of a preliminary *k*-means clustering step.^299^ In ref (300), a “divide-and-conquer” strategy has been proposed to automatically detect the cluster centers. Improvements on the estimation of the density have been addressed with kernel-like^301^ and *k*-NN-like approaches^302−304^ (see section 4).

In ref (305), the authors of the original method adopt a different approach to address many of its drawbacks. In short, the idea is to use the adaptive *k*-nearest neighbor estimator introduced in ref (13) for computing the density at each configuration. Then, a configuration is considered a possible density peak if its computed density is the highest within its neighborhood (the neighborhood is automatically defined by the algorithm as *k̂*_*i*_; see section 4.2.3). However, these density peaks may be a result of statistical fluctuation. This is addressed by using the error associated with the density estimation as follows: a density peak is considered “genuine” if the difference between the density at the maximum and the density at the saddle point is greater than *Z* times the sum of the errors of both estimations. *Z* is the only parameter of the method and is a measure of the statistic significance of the peaks. In this approach, all the possible saddles are first located as density maxima at the borders among peaks. Then, those peaks that do not pass the test of statistic significance are lumped together. This saddle analysis provides as additional feature a *topography* of the data set; namely, a tool that permits the hierarchical relationships between clusters to be considered.

The density peaks method has been optimized for the analysis of MD simulations,^306^ and with some modifications, the analysis of enhanced sampling MD simulations.^307^ Density peaks has been successfully employed for extracting binding poses from protein simulations^308^ and analyzing the type of sites involved in the jump of diffusing mobile ions in solid state simulations.^309^

It has been recently shown^180,297,305,310^ that if one uses a density-based clustering approach to find the peaks of a probability density estimated in a high-dimensional feature space, these peaks correspond very closely to the so-called “Markov states” of a molecular system, which will be introduced and discussed in section 6. The procedure described in these references effectively bypasses two of the steps, which are normally followed in the derivation of a Markovian model, which will be discussed in the next section: the dimensionality reduction from a large feature space *x* to a more compact representation *y*, and the clustering performed using one of the approaches described in sections 5.1.2 and 5.1.1. The drawback of the density-clustering based procedure is that the Markov states are an output of the clustering, and one can not attempt improving them, following the protocol described below in Section 6. One can only verify a posteriori if the states define a Markov model by estimating a transition probability restricted to these states, and verifying if the implied time scales are independent of the time lag. Exploiting these techniques together with density-clustering is a possibly interesting research line.

## 6 Kinetic Models

When running MD simulations, one does not generate statistically independent data points from the equilibrium distribution but rather *trajectories* in which the configurations are time ordered and in general correlated with each other. The information embedded in the time ordering can be exploited to perform an effective and grounded dimensionality reduction, and the resulting model can be used to extract kinetic information, such as transition rates, pathways, and time-correlation functions.

The inclusion of time correlations in the dimensionality reduction techniques described in section 3, leads to time-lagged independent component analysis and related linear methods (section 6.1), whereas the clustering framework described in section 5 leads to Markov state modeling (MSM) (section 6.3). Both methods can be unified under a “variational approach” (section 6.2), a framework, which can also be extended to nonequilibrium simulations (section 6.4) and can be used to obtain neural network representations (section 6.5).

An MD simulation can be formally described by the dynamical operator  that propagates the probability density of the system at state *t*, ρ(**x**_*r*,*t*_), to that of the system at time *t* + τ One can write
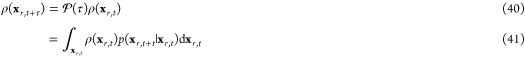
where *p*(**x**_*r*,*t*+τ_|**x**_*r*,*t*_) is the conditional probability density of finding the system at state **x**_*r*,*t*+τ_ given that it was at state **x**_*t*_ a number of time steps τ before. Note that eq 40 is a purely formal definition as the transition density *p*(**x**_*r*,*t*+τ_|**x**_*r*,*t*_) which usually cannot be explicitly computed. This propagator view is still extremely useful, however, as it is the basis for the development of the kinetic models and algorithms that we will describe here.

One can also define the matrix **P**(τ) as the discretized version of the propagator in eq 40. In the context of MSMs, for example, **P**(τ) contains the conditional transition probabilities between disjoint sets, or clusters, of state space. When modeling using the view of the propagator and its discretized erpart, the unknown quantities relating to the true physical system are approximated by known quantities obtained from the data. Throughout this section, we will use a “hat” to indicate the estimated or approximate quantity when the distinction is important.

### 6.1 Time-Lagged Independent Component Analysis

Time-lagged independent component analysis (TICA) was originally developed in the field of signal processing.^311^ For a time series of *T* ordered configurations {**x**_*t*_}, we can define the covariance matrix (**C**_00_), as well as the time-covariance matrix at “lag time” τ (**C**_0τ_) as
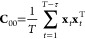
42
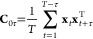
43where τ should be sufficiently small to resolve the dynamics of interest. The generalized eigenvalue problem that characterizes TICA is then given by,
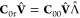
44where the eigenvalues λ̂_α_ are contained in the diagonal of **Λ̂**. Each eigenvector **v̂**_α_ is a column of **V̂** and characterizes a latent coordinate with maximal autocorrelation, which is defined as,
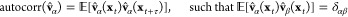
45As will be shown in section 6.2, TICA can be seen as a special case of the variational approach of conformation dynamics for time-reversible dynamics in equilibrium and for linear bases. Under these conditions, the time-covariance matrix **C**_0τ_ is symmetric (for *T* → *∞*),^312^ and as a consequence, the eigenvalues in eq 44 are real valued.

To guarantee that also at finite time *T*, λ̂_1_ = 1 and λ̂_2_ < 1 are achieved, one can use a symmetrized estimator for matrices, **C**_00_ and **C**_0τ_, as described in ref.^313^

The latent coordinates {**v̂**_α_} are the (orthogonal) slow processes within the dynamical system obtained from linear combinations of the degrees of freedom in {**x**_*t*_}. An estimate of the relaxation time scales of these processes can be obtained from their corresponding eigenvalues and the lag time according to
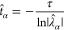
46TICA can be used for dimensionality reduction by analyzing the eigenvalue spectrum and truncating the basis {**v̂**_*i*_} at an observed spectral gap. Alternatively, one can employ kinetic mapping^131^ or commute mapping,^132^ which involve weighting the TICA components by their eigenvalues or time scales, respectively. These approaches are similar to the definition of diffusion distance (recall eq 14) in the context of diffusion map (see section 3.2.3), but in this case the low-dimensional manifold is embedded into a space in which geometric distances correspond to times required to transition between pairs of points.^131,132^ A reweighting procedure has also been recently developed to remove the bias of TICA estimates that arise from nonequilibrium sampling. While TICA is a linear method, it can be kernelized to accommodate nonlinear coordinates^314,315^ (see the treatment of Kernel PCA in section 3.2.2).

TICA was first used for molecular data to identify slow modes in MD simulations of proteins.^316^ Shortly after, it was employed as a preprocessing step in the construction of MSMs (see section 6.3).^252,317^ TICA has been leveraged to analyze a variety of biomolecular systems from both simulation and experimental data including the dynamics of protein folding,^253^ disordered proteins,^318^ protein–peptide, and protein–protein association,^29,256^ protein conformational change and ligand binding,^319^ binding-induced folding,^257^ and kinase functional dynamics^258^ TICA has also been integrated into enhanced sampling algorithms.^320,321^

### 6.2 Variational Approach to Conformational Dynamics

The Variational Approach to Conformation dynamics (VAC)^322^ is a principled approach to estimate the eigenvalues and eigenvectors of the dynamical propagator .^14^ Using VAC theory, it was shown that for a given feature representation **x**_*t*_ = **χ** (**x**_*r*,*t*_), the TICA algorithm produces the variationally optimal approximation to the long-time scale dynamics of .^317^

Specifically, the long-time dynamics is governed by the largest eigenvalues *λ*_*α*_ and eigenfunctions *ψ*_*α*_ of the dynamical propagator, for which corresponding relaxation time scales are given by eq 46.^14,19,21,323^ At this point it is convenient to consider not directly the propagator , but the so-called transfer operator ,
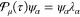
47which propagates probability densities that are normalized by the equilibrium density.^14^ This technical point goes beyond the scope of the current review; for now, it is sufficient to say that  and  both encode the system dynamics, and that for time-reversible dynamics we can easily switch between the two operators and their respective eigenfunctions (the interested reader may refer to ref (21) for details).

The aim of the VAC framework is to approximate the leading eigenvalue *λ*_*α*_ and eigenfunction *ψ*_*α*_, and in this sense it has a similar aim as the variational approach in quantum mechanics.^324^ Suppose we want to do that by a linear superposition of feature functions:
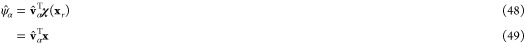
Given the covariance matrices defined in eqs 42 and 43), we define the discrete propagator:
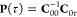
If one chooses indicator functions as features (i.e., **x**_*i*_ is 1 when **x**_*r*_ lies in the *i*th cluster and 0 otherwise), the matrix **C**_00_ simply contains the counts of the number of data points in each state, the matrix **C**_0τ_ is a transition count matrix and **P**(τ) is a transition probability matrix. For other types of features, **P**(τ) is a so-called Koopman matrix.^313,325^ One then performs an eigenvalue decomposition of **P**(τ):
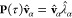
50According to VAC, the eigenvalues λ̂_α_ are best approximations to the true eigenvalues *λ*_*α*_ and ψ̂_α_ are best approximations to the true eigenfunctions *ψ*_*α*_ within what the linear superposition of feature functions (eq 48) allows. Note that this statement is true in the absence of statistical errors, namely, in the limit *T* → *∞* in eq 43.

Specifically, this solution maximizes the VAC-*r* variational score for the first *d* eigenvalue-eigenvector pairs, which is bounded from above by the VAC-*r* score of the highest *d* eigenvalues of the operator :^326^
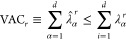
51where the equality only holds when the approximation is exact. VAC also implies that the approximated eigenvalues underestimate the true eigenvalues. By virtue of eq 46, this means that, in the limit of infinite data, a data-driven approximation to the true system may predict dynamics that are too fast, but never too slow.^327^ However, the approximation can be excellent for practical purposes when a sufficiently long lag time τ can be chosen.^328^

Because the VAC defines a scoring function, it enables us to optimize the model’s description of the true system’s global kinetics by variationally maximizing eq 51. For a given basis set used to represent {**x**_*t*_}, the procedure above is equivalent to TICA, and the TICA eigenvalue problem (44) yields the optimal linear approximation to {*v*_*α*_}.^317^ However, the existence of a variational score allows us to go beyond linear models and instead parametrize e.g., kernel or deep neural network models by interpreting eq 51 as a loss function.

If, instead, one needs to compare different basis sets for the same τ and *d*, the variational score (eq 51) can be used to choose the best basis set.^38,40^ Since the data available will always be finite, cross-validation should always be used when performing such comparisons,^38,40,329,330^ which is possible by exploiting the scalar score in eq 51. The choice of τ defines the particular propagator and its matrix approximation. To this point, we have only indicated that τ must be sufficiently small to resolve the dynamics of interest. When TICA or the VAC approach are employed in the context of Markovian dynamics (see section 6.3 below), τ should also be chosen to be sufficiently *large* so that the dynamics are indeed Markovian, or “memoryless”. This is discussed further in section 6.3.

The variational approach has been expanded to interpretations involving kinetic variance,^131^ commute distances,^132^ and diffusion mapping.^142^ Basis sets designed to be variationally optimized have also been developed,^331,332^ and a deep learning approach to optimize this loss function was reported in ref (333). This method of variationally optimizing a basis is crucial to modern MSM construction, which is discussed in the following section.

### 6.3 Markov State Modeling

In section 6.1, we discussed a strategy for reducing the dimensionality of a molecular trajectory data set {**x**_*t*_} to a set of coordinates that optimally describe the relaxation process of the system In practice, this may involve reducing hundreds or thousands of spatial degrees of freedom to only a few coordinates.

A different and more drastic way of compressing the information on a trajectory is to assign each configuration to a finite number of groups, as seen in section 5). If the groups represent “states” of the system, then this kind of clustering is called a Markov state model (MSM).^22^ A state is defined by an indicator basis functions that return 1 if and only if the system is in the corresponding state. With this choice, the covariance matrices **C**_00_ and **C**_0τ_ become matrices and the propagator **P**(τ) becomes a transition probability matrix (section 6.2). It was already understood by Zwanzig that a transition matrix **P**(τ) between metastable sets serves as an accurate approximation of the kinetics between these sets when the chosen lag time τ is longer than the time required to relax within the states.^334^

More recently, it has been proven that in a meaningful MSM **P**(τ) must approximate the leading eigenvalues and eigenfunctions of .^328^ This was an important step forward, as it confirmed that MSM state definitions do not need to be metastable: The MSM approximation error of a metastable state decomposition can be reduced by using a finer state discretization whose individual states are not metastable but give rise to a better approximation of the eigenfunction. It is especially important to have a finer discretization in the transition regions between two metastable states, where the slow-process eigenfunctions change a lot. This led to a tendency of constructing MSMs with many states. With the introduction of the VAC and TICA into MSM theory (see section 6.2), a more systematic approach was available to approximate the leading eigenfunctions of  using less states, and the MSM quality significantly improved.

From a set of state assignments, an MSM is constructed by counting the pairwise transitions at a lag time τ and storing those counts in a matrix. Using eq 40, this observed matrix is converted into a transition probability matrix, where each row sums to 1 and represents the discrete probability distribution of transitioning from the state at the row index to any other state including itself. An alternative approach to using a transition probability matrix is estimating a transition rate matrix,^19,335^ whose appeal is that it can propagate the modeled dynamics in continuous time instead of discrete time steps τ. Note, however, that neither strategy can resolve the fast part of the true dynamics (typically faster than τ), as MSMs and other master equation models coarse-grain the dynamics in space and time.

One typically wants the transition probability matrix to be not only row-stochastic but also to model a system that is ergodic and at thermodynamic equilibrium. To ensure ergodicity, every state must be accessible from every other state given a long enough simulation time. The system is not ergodic if different regions of its representation space are not kinetically connected. In that case, a subset of the system must be used which is locally ergodic. In practice, this is selected by identifying the largest connected subgraph of the graph implied by the transition count matrix **C**_0τ_.

Time-reversibility can be imposed on a reversibly connected transition matrix **P**(τ) via the detailed balance condition,
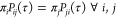
52Here, **π** is the vector of equilibrium probabilities, that is, **π**^T^**P** (τ) = **π**^T^. In other words, the equilibrium flux from any state *i* to *j* must be equal to the equilibrium flux from state *j* to *i* for all states. This also implies that there are no cycles in the flux. A great deal of research has gone into enforcing this constraint, which is not automatically satisfied when applying direct transition estimation to a finite simulation trajectory. Several studies have derived unbiased estimators for reversible MSMs (i.e., ones that obey detailed balance).^19−21,336,337^ An unbiased estimator for a reversible TICA model has also been recently introduced.^313^

The resulting transition matrix is a special case of the **P** matrix discussed in section 6.2, and the VAC can be applied to its eigendecomposition when detailed balance is obeyed. Although the approximated eigenfunctions {**v̂**_*i*_} are step functions in feature space, MSMs can have great expressive power because the feature transformations from **x**_*r*_ to **x**, where MSM states are defined, can be nonlinear.

As noted above, the lag time τ must be sufficiently large to have a good MSM approximation, while being small enough to resolve states of interest–see ref (338) for a mathematical analysis of this trade-off and ref (327) for a qualitative discussion. To check whether the MSM with lag time τ is indeed a good approximation of the long-time dynamics, we can assess its adherence to the Chapman–Kolmogorov property^16^
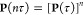
53In practice, to determine a suitable lag time τ one often first conducts a so-called “implied time scales” test by observing if the time scales in eq 46 converge to a constant as a function of the lag time.^16^ One can easily prove that if the time scales in eq 46 do not depend on τ, then the Chapman–Kolmogorov property holds.^16^

Practically, a straightforward MSM construction protocol proceeds roughly as follows:

1. Transform the spatial coordinates obtained from a simulation data set into a set of features, such as contact distances or dihedral angles (see section 2).
Ideally, these features capture symmetries in the system (e.g., roto-translational invariance).
2. Optionally perform a basis set transformation of the features, for example as by applying TICA (section 6.1).
The use of TICA as a preprocessing step for MSMs facilitates kinetic proximity within the states.^252,317^
3. Perform clustering (see section 5) on the data set to obtain discrete, disjoint states.
4. Construct the observed counts matrix and estimate a transition probability matrix for a chosen lag time, often using detailed balance constraints. The transition probability matrix and the state space decomposition define the MSM.
5. Assess the validity of the Markov assumption at that lag time using implied time scales or the Chapman–Kolmogorov test.

Applying the VAC (section 6.2) to MSM construction entails performing the steps above for the same lag time and assessing its variational score (as defined in eq 51) for the same number of eigenvalues. This must be done under cross-validation, that is, by performing multiple instances of fitting the MSM to a training set and evaluating it on a held out test set.^329^ This procedure is discussed in detail in ref (38). For more detailed reviews of the theory underlying MSMs, we refer the reader to refs (21), (22), and (339).

The number of microstates (clusters) in an MSM, following the variational optimization of its parameters is often to large to lead to a readily interpretable model. For MSMs constructed from data sets with millions of points in time, hundreds of microstates are often found to be appropriate.^38,278^ The further coarse-graining of MSM state space into so-called “macrostates” has been part of the MSM construction pipeline since the first analyses of small peptide and protein folding systems.^17−19,340,341^ Indeed, algorithms to find macrostates have been developed alongside MSM methods since the first formalizations of the latter.^14,342^ A summary of these methods and pertinent references are in ref (22).

**Figure 7:**
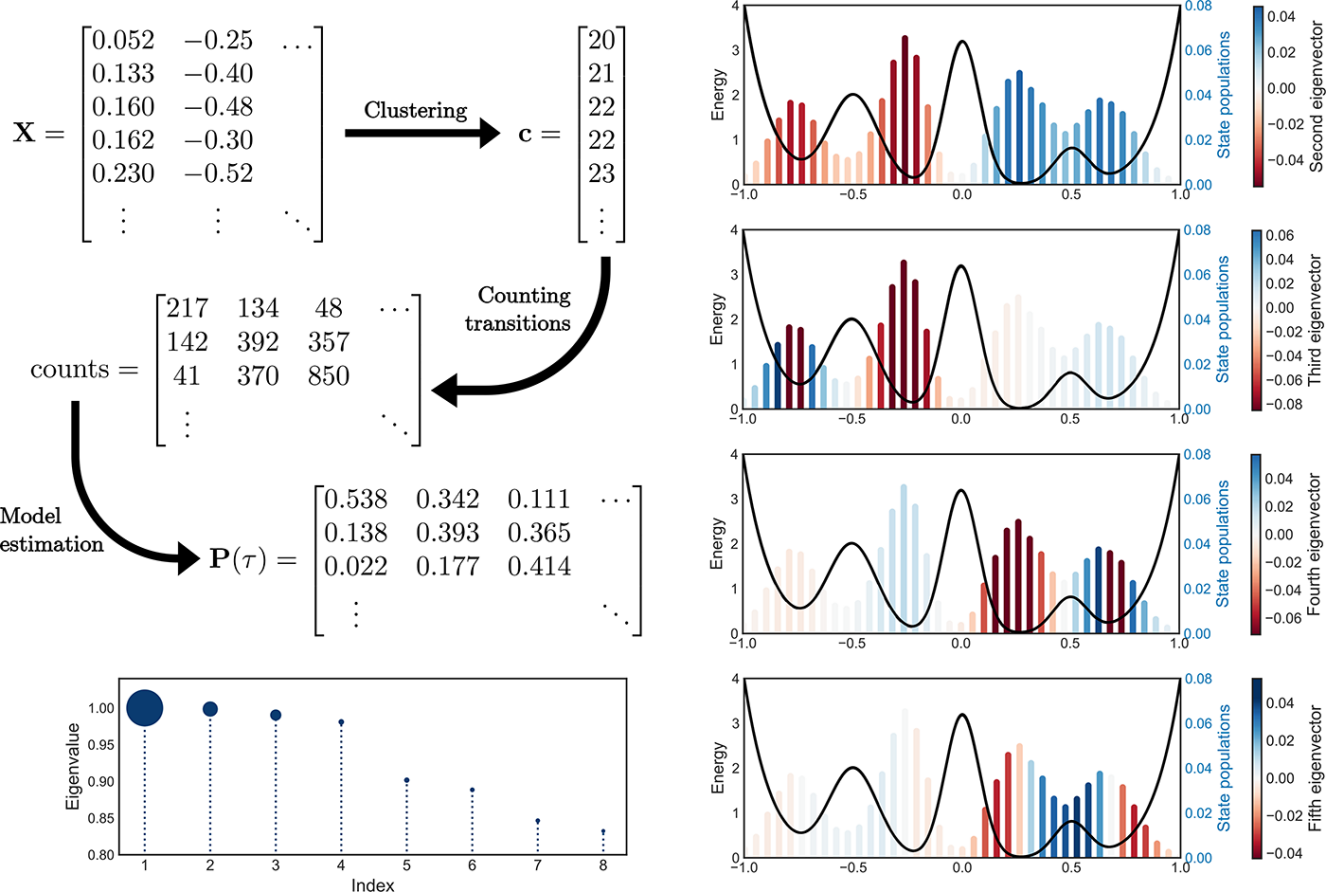
Schematic illustration describing the MSM construction. First, the data **X** is reduced to a sequence of integer states **c**. Then, transitions among those states after a duration of a lag time τ are counted and stored in a count matrix. Next, the MSM itself is estimated from the count matrix to create a transition matrix **P**(τ). The eigenvalues of **P**(τ) correspond to the time scales of the process according to *t*_*α*_ ≡ −τ/log|λ̂_α_|. The sizes of circles in the bottom left plot correspond to time scale magnitudes; it can be observed that small changes in eigenvalues result in large changes in time scales due to the logarithmic transformation. On the right, the state populations are shown for the first four dynamical eigenvectors (corresponding to eigenvalue indexes 2–5), along with the underlying potential. The heights of the bars indicate state populations, and the colors indicate flux into (blue) and out of (red) various states.

Given a valid MSM, the eigenvalues and eigenvectors of **P**(τ) can be analyzed and interpreted. According to the Perron–Frobenius theorem, the dominant eigenvalue is 1 and its corresponding eigenvector contains the equilibrium populations of the states. Every subsequent eigenvalue is smaller than 1 and its corresponding eigenvector represents a dynamical process characterizing flux among the MSM states. The time scales of these processes can be determined from their eigenvalues according to eq 46. A schematic illustrating of the MSM construction for a one-dimensional potential^21^ is provided in Figure 7. As MSMs reveal the long-time relaxations, they are naturally well-suited to compute observables of kinetic experiments, such as time-correlation spectroscopy.^19,323,343^

MSMs have proven to be of great use to various applications in studies of molecular—particularly biomolecular—kinetics. These includes the analysis of biophysical processes, such as peptide dynamics^17−19,336,344−356^ and their oligomerization,^357^ protein folding,^277,340,341,358−366^ or a simplified model thereof,^367^ ligand binding,^368−374^ and conformational change.^258,375−377^ Dynamics of other processes, such as carbon nanotubes^378^ and solid state atomic systems,^379^ were also described using MSMs.

Importantly, MSMs can also reveal the mechanisms of transitions between long-lived states. Transition state theory, originally developed in ref (380), was formulated for MSMs and master equation models in refs (255 and 351) and first applied in ref (255) to compute the full ensemble of protein folding paths of a miniprotein. Similar analyses were conducted for ligand binding,^381,382^ kinase activation,^383^ and protein–protein association.^29^

Whereas the methods described to this point rely on the time-ordering of the data set, some of the density peaks clustering methods described in section 5.2.2 can produce MSMs without requiring time information nor explicit dimensionality reduction or clustering steps.^180,297,305,310^ While this approach is powerful for the above reasons, one drawback of the density peaks approach to MSM construction is that the Markov states are an output of the clustering; thus, one can not attempt to improve them variationally as can be done with the standard protocol described above. Instead, one can only verify a posteriori if the states define an adequate MSM by estimating a transition probability matrix restricted to these states and, subsequently, examining the implied time scales.

### 6.4 Koopman Models and VAMP

The algorithms described in sections 6.1, 6.2, and 6.3 require the use of reversible data that obeys the detailed balance condition (eq 52). However, this assumption of microscopic reversibility is not appropriate in all cases. For example, some simulations are deliberately performed out of equilibrium, such as studies of membrane channel conductivity in an external electrostatic potential, or when simulating a force-probe experiment.

To deal with both equilibrium and nonequilibrium data, one can use Koopman models^313,325^ and the variational approach to Markov processes (VAMP), which is a generalization of the VAC.^330,384^ In the nonequilibrium analogue of TICA one estimates the same covariance matrices as in TICA, and additionally
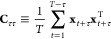
54which is an instantaneous covariance matrix as **C**_00_ but at the time-point *t* + τ. If dynamics are stationary, that is, the dynamical propagator does not depend on the absolute value of the time, we expect **C**_00_ = **C**_*ττ*_ in the statistical limit, but if the molecular system is driven by an external force, that is, as in force-induced protein unfolding, or if the simulation trajectories are simply too short to have equilibrated, we will have **C**_00_ ≠ **C**_*ττ*_ even in the limit of infinitely many simulation trajectories. Then, the variationally optimal latent coordinates are obtained by a singular value decomposition of a modified propagation matrix
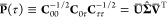
55where the latent coordinates {**u**_α_} for the data are stored in the columns of , the latent coordinates {**v**_α_} for the time-lagged data are the columns of , and the singular values σ̂_α_ comprise the diagonal of **Σ̂**.^259,330^ Note that the singular value decomposition in eq 55 reduces to a standard eigendecomposition when **C**_0τ_ is symmetric.

The linear VAMP described above—that is, the nonreversible analogue of TICA—is also called time-lagged canonical correlation analysis, or TCCA.^4,330^ For reversible data, eq 55 is equivalent to eq 44.^259^ Furthermore, MSMs are a special case of Koopman models when the data is both reversible and represented by a basis of indicator functions.^330^ The interested reader is further referred to ref (259), which demonstrates the equivalence of TICA and TCCA under reversible conditions, as well as the relationship of both algorithms to CCA in general.

Importantly, like the VAC, VAMP defines a variational score that can be used to optimize arbitrary shallow or deep machine learning architectures to obtain MSMs or Koopman models for equilibrium or nonequilibrium data:^330^
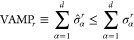
56where σ̂_α_ are the data-driven estimates of the singular values *σ*_*α*_ of the true dynamical operator .^330^

**Figure 8:**
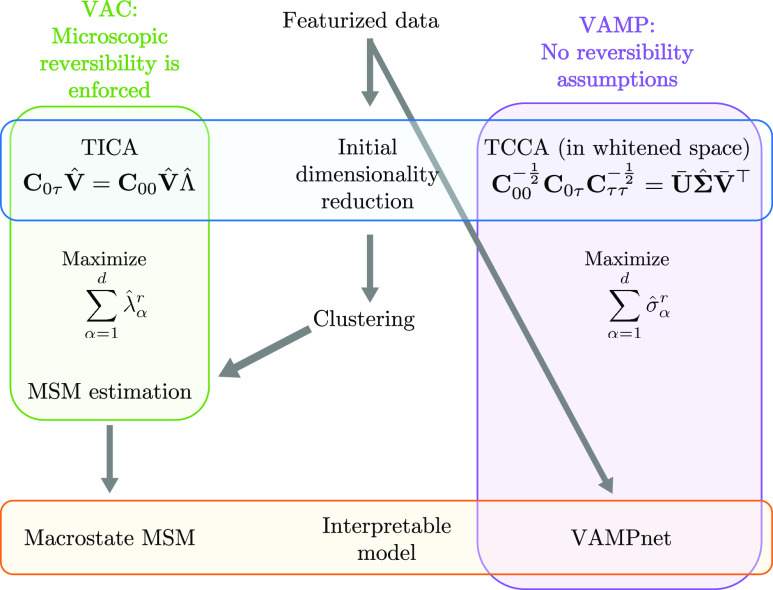
From featurized data, an analysis of molecular kinetics can proceed under the assumption of microscopic reversibility (i.e., equilibrium) or not. In the former case, TICA can be applied to reduce dimensionality and may serve as a final or intermediate model. When TICA serves as a step in the MSM construction pipeline, clustering is performed in TICA space and the MSM is estimated from the cluster assignments. Both TICA and MSMs adhere to the VAC, which acts on the eigenvalues of the propagator approximation. A macrostate MSM can be created if further interpretability is desired. In the latter case (no reversibility assumption), TCCA is used instead of TICA to reduce the dimensionality of the data set. From there, clustering can be performed and a reversible MSM can be constructed, or the TCCA results can be regarded as the final model. The relevant variational principle is VAMP, which acts on the singular values of the propagator approximation. A VAMPnet bypasses the majority of the construction pipeline by creating an interpretable model directly from featurized data. VAMPnets employ the VAMP criterion as a loss function in a neural network scheme.

Such a procedure is described in ref (40) and is used to perform feature selection in protein folding. The application of Koopman models to the dynamics of an ion channel in the presence of an electrical gradient is demonstrated in ref (35). The optimization of the VAMP score (eq 56) with neural networks has been presented in ref (158) and is briefly discussed in the following section. Figure 8 depicts the relationships among the variational algorithms discussed in Sections 6.1–6.4.

### 6.5 VAMPnets

In section 6.1, we began with TICA, a dimensionality reduction method that incorporates the temporal ordering of a data set to obtain a low dimensional embedding that best preserves the slowest dynamical processes in the data. Then, in section 6.2, we described the VAC, a framework for variationally approximating the slow dynamical modes of the system. The VAC framework has two key implications. First, it allows us to interpret the TICA embedding as the best variational approximation of the system’s slow mode for a linear basis. Second, it allows us to compare different bases, as the VAC score can be used to assess the quality of the resulting model.

In section 6.3, we discussed Markov state modeling, a VAC-compatible method that employs an indicator function basis to partition a data set into discrete, disjoint states. The VAC enables various parameters involved in Markov state modeling (e.g., feature choices, clustering method, and number of clusters) to be compared to determine the parameter set that best approximates the dynamics in the true data.

Sections 6.1–6.3 are designed for reversible data; that is, assumed to be sampled from an equilibrium ensemble. Section 6.4, then, demonstrates that TICA and the VAC can both be generalized to accommodate nonreversible dynamics through TCCA and VAMP, respectively (note that TCCA is equivalent to the linear VAMP). Together, these advances led to the development of VAMPnets:^158^ a machine learning framework that leverages the variational approach to learn a kinetic model of the data by optimizing a loss function.

It was first proposed by McGibbon and Pande^329^ that the variational score introduced in eq 51 can be interpreted as a loss function and, therefore, can be used to optimize, for example, the parameters of a MSM. Particularly, McGibbon and Pande advocated for the use of cross-validation when determining hyperparameters to accommodate for the necessarily finite sample size as the variational principle is only exact for infinite data.^329^ In the generalization of the reversible VAC (eq 51) to the nonreversible VAMP (eq 56), Wu and Noé also focused on the need for cross-validation under variational optimization of hyperparameters.^330^

**Figure 9:**
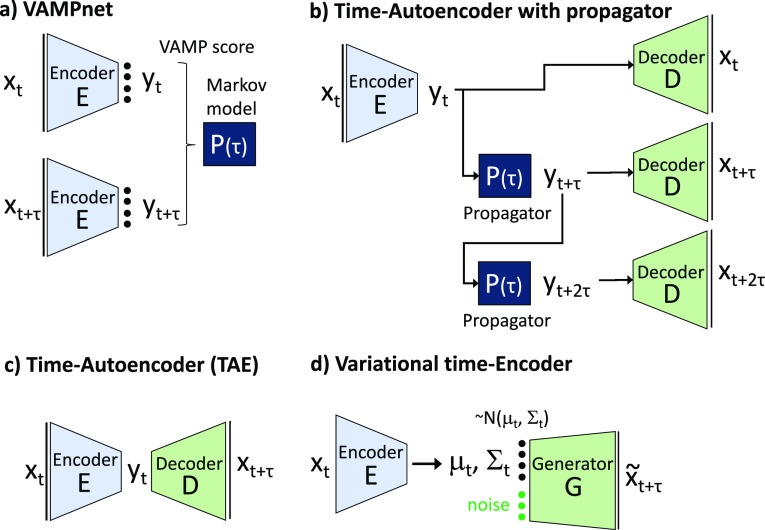
Overview of network structures for learning Markovian dynamical models: (a) VAMPnets,^158^ (b) time-autoencoder with propagator,^387,388^ (c) time-autoencoder,^160^ and (d) variational time-encoder.^159^

With VAMPnets,^158^ this insight enables the replacement of the Koopman model construction pipeline with a neural network architecture. It has been shown that constructing a reasonable MSM, following the pipeline described in section 6.3 requires a great deal of trial-and-error.^38−40,278,385^ To avoid extensive hyperparameter searching in such a sequence of steps, VAMPnets purport to use a self-supervised neural network architecture to collapse the construction procedure (steps 1–4 for MSMs as enumerated in section 6.3) to a single step, in which the VAMP score (eq 56) is used to optimize the featurization network and Markov or Koopman model in an end-to-end fashion (Figure 9a). With VAMPnets, the user only needs to provide the initial features and the neural network learns a deep feature representation of a low-dimensional latent space that approximates the eigenfunctions (or singular functions) corresponding to the slow dynamical processes. In this space, a Markov state model or Master equation model of the molecular kinetics is readily obtained. The pipeline is fully automated when the input features are the “raw” simulation data (i.e., Cartesian coordinates); alternatively, features can be determined in a preprocessing step. Several related architectures have been developed which are schematically presented in Figure 9.

VAMPnets have proven useful on protein folding data sets.^158^ Similar work on protein folding has followed that employs the VAMP basis function in an autoencoder framework^159,160,162^ (Figure 9c and d).

The VAMPnet architecture has also been recently used to analyze functional materials.^386^ Similar architectures have been developed to learn deep dynamics models of fluid mechanical systems in an end-to-end fashion^387,388^ (Figure 9b).

## 7 Relevant Software

### 7.1 Feature Representations

Among many other tools, the *PyEMMA*(385,389) Python library contains the construction of standard geometric and knowledge-based features for biomolecular systems, particularly protein systems. The *MDTraj* Python library^390^ (which is internally used by *PyEMMA*) is also a resource for creating feature representations from MD simulation data. *PLUMED*(25) allows computing a very large number of collective variables, and even defining ad hoc functions. The three packages *DScribe*,^63^*PANNA,*^391^ and *Librascal*(392) can be used to compute all the most widely used numerical features for condensed matter systems.

### 7.2 Dimensionality Reduction

*Scikit-learn*,^393^ which itself sources from *NumPy*,^394^*SciPy*,^395^ and *Matplotlib*,^396^ is a widely used statistical learning package that encompasses a large number of unsupervised learning methods and tools for supervised learning. Linear dimensionality reduction methods like PCA and MDS are included in the package, along with more complex and nonlinear methods like Isomap, Kernel PCA, and t-SNE. The Diffusion Maps method can be found in the *pyDiffMap* package, available at ref (397). An implementation of the original Sketch-map algorithm^143^ can be downloaded from ref (398). Algorithms of deep manifold learning (such as deep autoencoders) are not implemented in any specific standard package, since there is no standard way of constructing such architectures. Libraries, such as *Pytorch*,^399^*TensorFlow*,^400^ and *Keras*,^401^ can be however used to quickly implement such deep learning architectures. The models are typically available as Supporting Information in the relevant references. For example, the time lagged autoencoder from ref (160) is available at ref (402).

### 7.3 Density Estimation

The *Scikit-learn* library also contains a variety of algorithms for density estimation. Parametric density estimators such as the Gaussian mixture model can be found there, along with nonparametric estimators, such as the histogram estimator and the kernel density estimator. The Point Adaptive *k*-NN estimator can be found at ref (403) or at ref (404).

### 7.4 Clustering

*Scikit-learn* also implements many clustering algorithms. Algorithms like *k*-means and its extensions, as well as DBSCAN, spectral clustering, and hierarchical clustering can all be found there. The *Deeptime* python library, available at,^405^ includes *k*-means clustering and some of its extensions. In *METAGUI3*,^307^ several clustering algorithms like *k*-medoids or density peaks clustering have been adapted to its use in the context of biased simulations. An implementation of the Advanced density peaks clustering can be found at ref (403) or at ref (404).

Various software packages have been developed that facilitate the construction of the kinetic models discussed in section 6. As mentioned above, *PyEMMA* contains tools for the featurization of biomolecules. In fact, *PyEMMA* was designed to provide an all-in-one tool to create a variety of kinetic models, including MSMs, from unprocessed simulation data. A variety of tutorials are available online and are described in detail in ref (389). The more recent *Deeptime* software provides a generalization of many of these methods beyond biomolecular systems, and performance improvements. Both *PyEMMA* and *Deeptime* can be used for full kinetic model creation, but both packages are written in a modular style that enables the use of a particular algorithm for just one step in kinetic model building. Thus, both packages can be used for dimensionality reduction, clustering, and transition matrix estimation without going through the entire construction pipeline. The *Enspara* library^406^ is an alternative library for Markov state modeling that provides specialized data structures to improve scalability to very large data sets.

## 8 Conclusion and Discussion

Following the increase in processing power and data storage capabilities of the last several decades, molecular simulations have now arrived at an unprecedented level of complexity. Millisecond long simulations of millions of particles are now relatively common, and simulations of more than one billion particles can be achieved on specialized computer architecture.^407,408^ In this work, we have provided a comprehensive overview of the unsupervised learning techniques that have proven to be the most useful for the analysis of molecular simulation data.

We started our overview in section 2 with a discussion on the various ways in which a trajectory can be encoded into a numerical representation. Here, the goal is to transform the “raw” Cartesian coordinates of the constituent particles into a more compact numerical representation that preserves all the *relevant* information on the trajectory. This procedure is a necessary prerequisite for performing any analysis on a trajectory data set, and it is of fundamental importance since it will affect any algorithm subsequently deployed on the chosen representation. For instance, an overly restrictive representation might lead to a systematic bias, while a representation that is too redundant might lead to an increase in computational cost.

The automatic choice of an informative yet compact data representation can be considered an open challenge. It is still common practice to exploit expert knowledge of the system in order to omit degrees of freedom that are deemed irrelevant for the scope of the analysis. For instance, many protein folding studies ignore solvent degrees of freedom since they are considered unimportant for defining the protein states. However, it is well-known that solvent does play a role,^409,410^ in simple chemical reactions as well as in complex biomolecular transitions, and researchers have developed dedicated collective variables capable of capturing these phenomena.^411−413^ In the spirit of unsupervised learning, ideally one would like to treat the “solvent” in an agnostic manner, at the same level of the “solute”, and automatically learn the most relevant features from data. We believe that substantial improvements are still possible in this direction.

A similar challenge can be identified in the fields of material science and solid state physics. Here, the guiding principle for choosing a data representation is to exploit the knowledge of the symmetries of the system, such as the invariance with respect to exchanges of identical atoms or to rigid rotations. The SOAP and ACSF features are built to automatically satisfy these properties, and represent prominent examples of data representation. However, this manner of representing the configurations becomes memory-intensive when the number of atomic species is more than 3 or 4. Moreover, the definition of these features involves the choice of some important hyperparameters, such as the size of the neighborhood used to compute the features: if this size is too small one risks to overlook important details in the medium-range organization of the system, while if the size is too large the number of features increases rapidly. Making SOAP and ACSF features definition more general and robust can hence be considered another important open challenge.

As a general guiding principle for choosing a feature space, unless very specific system knowledge is available, it is not advisable to select by hand a very small set of coordinates to describe the system, since any data representation can be made more compact using specifically designed methods of *dimensionality reduction*.

In section 3, we reviewed the algorithms which can be exploited to capture the structure of the data manifold as it appears in the original space using a lower-dimensional representation. The most important and well-known approach to perform this task is PCA, which has been extensively used to analyze molecular simulations for many decades, well before any other unsupervised learning method. This approach is computationally efficient, it is grounded on a robust and simple theory, and it is exact if the data manifold coincides with or is contained in a hyperplane. Several alternative methods have been developed which generalize PCA to the case in which the data manifold is curved and twisted. The most prominent examples are Isomap and Kernel-PCA, which allow “ironing” a curved manifold.

The topology of the embedding manifold poses a hard constraint upon the maximum level of dimensionality reduction which can be achieved with these methods. Consider, for example, a case in which the data points lie on the surface of a three-dimensional sphere. Even if the manifold is two-dimensional, it is impossible—in theory and in practice—to find a two-dimensional representation of the data that preserves the neighborhood relationship of all the data points. In other words, if the embedding manifold is not topologically equivalent to an hyperplane, it is impossible to reduce the dimension of the representation up to the “natural” threshold, which would be the intrinsic dimension of the data.

The front end of research in this field is the development of approaches capable of performing a dimensionality reduction in highly nonlinear and topologically complex manifolds. A remarkable attempt in this direction is the Sketch-map approach, which was developed specifically for visualizing and analyzing molecular simulations. This approach has in principle the potential to go well beyond PCA and Kernel-PCA, but at the price of abandoning the simplicity of linear algebra. Indeed, in the Sketch-map approach the low-dimensional representation is found by optimizing a highly nonlinear functional, which moreover depends on several hyperparameters whose values are system-dependent. Other recent approaches to perform nonlinear dimensionality reduction in molecular simulations are based on neural networks. As discussed in section 3.2.6, autoencoders are a natural choice to address this task. We believe that these approaches can still be significantly improved, for example by porting the impressive know-how that has been developed in image recognition and language processing to the molecular world. For instance, state-of-the-art networks for image recognition are typically very deep and strongly overparameterized, and it is now well understood that this choice helps developing robust and transferable models. This change of paradigm has been so far only partially digested by the atomistic simulation community, where it is still common to exploit architectures with very few hidden layers, and with relatively few parameters.

Another key tool for recapitulating the results of a molecular simulation is estimating the probability density or, equivalently, the free energy (section section 4). Density estimation is a topic that has received much attention in data science.^202^ In molecular simulations, choosing the best density estimation method is highly nontrivial, especially if one aims to estimate the probability density as a simultaneous function of many coordinates. Moreover, the probability density in a system characterized by the presence of metastable states varies by several orders of magnitude *by definition*. If information on a plausible form of the density distribution is available, a viable option is estimating the free energy by a parametric method (e.g., mixture models). An alternative strategy, which we described in detail in this review, is estimating the probability density directly on the embedding manifold; that is, without performing any explicit dimensionality reduction beyond the choice of the features describing the system.^13^ An open challenge in the field is the development of a free energy estimator, which can be used in high-dimensional feature spaces and, at the same time, can provide an explicit and differentiable function of the coordinates. An important first step forward in this direction has been made, once again using neural networks,^414^ where the NN is optimized to return the value of the free energy and its gradient as a function of many collective variables.

In section 5, we review the clustering algorithms that are most commonly used for analyzing molecular simulations. Clustering can be seen an unsupervised dimensional reduction technique, in which a multidimensional data landscape is mapped to a finite set of states. The use of clustering in the analysis of molecular simulations has been ubiquitous since the seminal work of Brünger et al.,^415^ but as highlighted in this Review, the interpretation of a set of clusters will be radically different for different modeling approaches. In *k*-means and related methods, the clusters correspond to a *partition* of the data landscape, which in some approaches approximates a Voronoi tessellation. This partition can be made as fine-grained as desired according to a fixed parameter, which, in the simplest case, is simply the number of clusters. In more advanced approaches, such as hierarchical methods or spectral clustering, the optimal clustering model can be inferred directly from the data, for example, by analyzing the structure of a tree in dendrogram-based approaches. Partitioning schemes are essential for inferring a dynamic model from a simulation since, as discussed in section 6, they provide an appropriate basis for estimating the transition probabilities. In density-based clustering the clusters have a one-to-one correspondence with probability maxima (or, equivalently, to free energy minima).

In section 6, we present on overview of algorithms for dimensionality reduction that explicitly exploit the time-ordering of a molecular dynamics trajectory. The key idea at the basis of many approaches is that the eigenvectors of the matrix approximation of the dynamical propagator describe the slow kinetic modes of a system, which are also assumed to be the most relevant. A straightforward, linear way to estimate these slow modes is through TICA, which has become a cornerstone of many kinetic analyses. In 2013, it was shown that TICA provides the *optimal* linear approximations to the leading eigenfunctions of the true dynamical propagator. This is a special case of the VAC, a variational framework that defines a scalar score for assessing the fidelity of the dynamical modes of a system.

The VAC is a powerful approach that enables the systematic optimization of a kinetic model. One kinetic modeling paradigm that greatly benefits from the development of the VAC is that of Markov state modeling (see section 6.3). The standard protocol for constructing a MSM leverages the featurization protocols described in section 2, the dimensionality reduction techniques discussed in section 3 and the clustering methods summarized in section 5. Alternatively, these steps can be bypassed by performing density-based clustering, which in principle directly provides the Markov states (see section 4), but without the benefits of the VAC. Although the construction of a MSM requires many hyperparameters related to the choice of featurization, dimensionality reduction, and clustering steps, the VAC enables these hyperparameters to be optimized in an objective fashion.

TICA, the VAC, and MSMs are designed for the dynamics of systems at thermodynamic equilibrium; that is, those that adhere to microscopic reversibility. Recently these approaches were extended to their nonequilibrium analogues—TCCA, VAMP, and Koopman models, respectively (see section 6.4). This suite of methods yield analyses that are less physically interpretable than their reversible counterparts, but can accommodate a greater range of chemical and biophysical systems. An important advance in this class of algorithms is their recent combination with the modern deep learning approaches briefly outlined in section 3.2.6. The key idea is that the VAC or VAMP score is interpreted as a loss function, and a deep neural network incorporating feature representation, dimensionality reduction and clustering can be optimized end-to-end using backpropagation. The combination of state-of-the-art kinetic modeling tools with deep learning is an exciting area for future methods development, and holds the promise of selecting model hyperparameters in a rigorous, automatic, and possibly transferable way.

Altogether, we have given an overview of unsupervised learning methods for data representation, dimensionality reduction, clustering and kinetic modeling in molecular simulation. We have primarily focused on the discussion of classical, so-called “shallow” machine learning methods in which the relevant statistics of the data are often collected in vectors or matrices and then a linear algebra method is solved (e.g., PCA, MSMs, diffusion map) or a simple algorithm is iterated (e.g., *k*-means). The result of the calculation represents the dimensionality reduction, clustering, or kinetic model and often allows us to conduct further analysis in a relatively straightforward way, such as trying to understand which molecular features are most relevant in a learned low-dimensional representation (e.g., PCA, TICA), or compute a variety of kinetic properties (e.g., MSMs). These shallow machine learning methods are robust, efficient, easy to implement and have withstood the test of time.

On the other hand, an overwhelming amount of recent research focuses on deep learning methods, triggered by the renaissance of these methods since the AlexNet paper on image recognition.^416^ Deep learning methods are notable in that they can exploit (and require) large amounts of data, and they can learn highly nonlinear transformations and hidden complex patterns that a human designer might not be able to come up with. Their downside is that they are very expensive to train, have a high memory consumption to store their many parameters and their predictions may be unstable and susceptible to noise unless care is taken to prevent that.

Whereas in traditional machine learning applications, such as image processing or game-playing, deep learning methods clearly define the state of the art, this is not always clear in scientific tasks such as the analysis of molecular simulations. As deep learning methods become more and more established in all areas of science, more emphasis should be placed on their efficiency and reproducibility rather than simply the application of a deep learning idea to a new problem setting. It is our opinion that shallow and deep learning methods both have a role to play in this consideration.
